# Tat-HSPE1 suppresses clear cell renal cell carcinoma growth through lysosome-dependent cell death

**DOI:** 10.3389/fphar.2026.1822208

**Published:** 2026-05-01

**Authors:** Lin Zhang, Weiyuan Li, Min Liu, Dong Li, Guang-Zhi Jin

**Affiliations:** 1 Tongren Hospital Shanghai Jiao Tong University School of Medicine, Shanghai, China; 2 Department of Urology, Shanghai Tenth People’s Hospital, Tongji University School of Medicine, Shanghai, China; 3 Department of Urology, Tongren Hospital Shanghai Jiao Tong University School of Medicine, Shanghai, China; 4 Department of Pathology, Tongren Hospital Shanghai Jiao Tong University School of Medicine, Shanghai, China

**Keywords:** autophagy, CTTNBP2NL, heat shock proteins, lysosomal membrane permeabilization (lmp), peptide, renal cancer

## Abstract

Clear cell renal cell carcinoma (ccRCC) is the most prevalent subtype of kidney cancer; however, first-line therapeutic agents show limited efficacy in patients with advanced disease. Bioactive peptides have emerged as promising candidates for anticancer therapy. In this study, a novel peptide derived from Heat Shock Protein Family E Member 1 (HSPE1) was identified by peptidomics analysis of human ccRCC tissues and paired adjacent normal tissues. We then engineered a novel fusion peptide designated Tat-HSPE1. Tat-HSPE1 selectively induced *in vitro* cell death in ccRCC cells while exerting minimal cytotoxic effects on normal epithelial cells and other tumor cell types. Analyses on the potential mechanism revealed that Tat-HSPE1 induced DNA damage, caspase-independent apoptosis, and lysosomal membrane permeabilization. Following cellular uptake, Tat-HSPE1 preferentially accumulated within the nucleolar compartment, where it interacted with CTTNBP2NL, a newly identified negative regulator of autophagy. This interaction promoted the translocation of CTTNBP2NL from the nucleus to the cytoplasm, facilitating the activation of autophagic processes. Furthermore, *in vivo* experiments demonstrated that Tat-HSPE1 significantly suppressed xenograft tumor growth. These findings indicate that Tat-HSPE1 represents a promising peptide-based therapeutic candidate for the treatment of ccRCC.

## Introduction

Renal cell carcinoma (RCC) is one of the most common malignant tumors. An estimated 430,000 new cases and 156,000 deaths were recorded globally in 2022, while the 5-year overall survival rate varied from 40% to 75% across different regions (Larcher et al., 2025). Clear cell renal cell carcinoma (ccRCC), the most prevalent subtype of RCC, is exhibiting a rise in global incidence and is associated with significant mortality. Over the past few decades, treatment for RCC has evolved markedly—from surgical tumor resection combined with high-dose cytokine therapy to targeted therapies, immunotherapies, and their combinations, and moving potentially towards individualized cellular therapies in the future. Despite these remarkable strides, approximately 30% of patients experience recurrence and metastasis following surgical intervention (Dell'Atti et al., 2022; Motzer et al., 2024). Current first-line therapeutic agents demonstrate limited efficacy in advanced-stage patients, with the 5-year survival rate remaining below 20% (Zhao et al., 2024), underscoring the urgent need to develop novel and potent antitumor compounds.

Research on peptide-based drug development and clinical translation is continuously expanding, offering distinct advantages in targeting precision and biological activity over traditional small-molecule therapeutics (Lv et al., 2021). As highly efficient and highly selective biomolecules with low toxicity, peptides play a pivotal role in cancer therapy, facilitating key biological processes such as precise targeted delivery, tumor penetration, and participation in biocatalytic pathways (Hamley, 2017; Muttenthaler et al., 2021). Therapeutic peptides can be designed to provide specialized functions based on distinct cell types, signaling pathways or tumor suppressor proteins, enabling the selective eradication of cancer cells while maximally preserving the integrity of healthy tissues (Zhang et al., 2019; Gao et al., 2023; Mukherjee et al., 2023). For instance, C16-(EY)3, a peptide amphiphile, undergoes phosphorylation within cancer cells and triggers endoplasmic reticulum stress. C16-(EY)3 can activate caspase-dependent apoptotic signaling pathways and selectively induce apoptosis in cancer cells overexpressing tyrosine kinases (Shimizu et al., 2025). In in vitro and *in vivo* colorectal cancer models, a short peptide Pep3S demonstrated robust anti-tumor efficacy by binding MDM2 with high affinity, effectively reactivating wild-type p53 in human tumors (Va et al., 2025).

Previously, we performed a comparative peptidomics analysis of human ccRCC and para-cancer tissues to explore differentially expressed endogenous peptides that may be involved in tumor progression. Based on the data obtained, we designed and synthesized a cell-permeable peptide named Tat-hspb1, which could inhibit the progression of ccRCC by inducing lysosomal membrane permeabilization (LMP), potentially serving as a new anticancer drug candidate (Zhang et al., 2022). Using the same peptidomics dataset, we selected a peptide derived from the heat shock protein HSPE1, together with several randomly selected peptides as preliminary controls. These sequences were conjugated to the cell-penetrating peptide Tat (RKKRRQRRR) to generate a series of fusion peptides. Among them, Tat-HSPE1 displayed pronounced cytotoxic activity against renal carcinoma cells, whereas the other peptides exerted minimal effects on cell viability.

In the present study, we demonstrate that Tat-HSPE1 markedly inhibits ccRCC cell growth by selectively inducing cell death in malignant cells without compromising normal epithelial cells and other tumor cell types. Tat-HSPE1 mainly localized to the nucleolus and interacted with CTTNBP2NL (Cortactin-binding protein 2 N-terminal like), a newly identified negative regulator of autophagy (Goudreault et al., 2008). Furthermore, Tat-HSPE1 induced DNA damage, caspase-independent apoptosis and lysosomal membrane permeabilization, ultimately leading to cell death. Collectively, these findings identify Tat-HSPE1 as a novel peptide-based therapeutic candidate and provide a potential new strategy for the treatment of ccRCC.

## Results

### Identification of Tat-HSPE1 as a candidate therapeutic peptide for ccRCC

To identify potential therapeutic peptides for ccRCC, we applied a peptidomics analysis of three pairs of ccRCC and adjacent normal kidney tissue samples, a detailed description and complete analysis of this work has been reported elsewhere (Zhang et al., 2022). These differentially expressed peptides originate from distinct precursor proteins. In our previous work, we synthesized the antitumor peptide Tat-hspb1 and identified the therapeutic potential of heat shock protein–derived peptides in ccRCC. Accumulating evidence indicates that heat shock proteins purified from specific tumors can elicit tumor-specific immune responses, highlighting the heat shock protein family as a highly promising focus in cancer therapeutics (Ménoret and Bell, 2000). In addition, HSPs have been implicated in multiple aspects of tumor biology, including cell survival, proliferation, drug resistance, and regulation of autophagy, suggesting that they function not only as key drivers of tumor progression but also as druggable targets for precision oncology. On the basis of these observations and our previous findings, we hypothesized that peptides derived from heat shock proteins may likewise exert critical regulatory roles in tumor cells.

**FIGURE 1:**
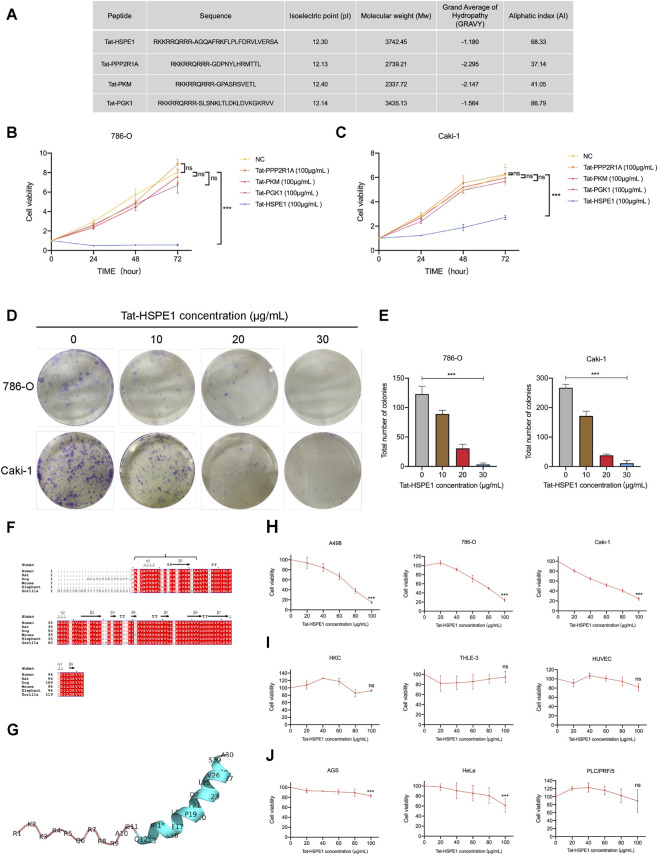
Tat-HSPE1 inhibits cell viability and proliferation of ccRCC. **(A)** Physical and chemical parameters of candidate peptides using Expasy (https://web.expasy.org/protparam/). **(B,C)** CCK-8 assay measuring cell viability of 786-O and Caki-1 cells treated with candidate peptides for 24, 48 and 72 h **(D,E)** Colony froming assay evaluating cell proliferation of 786-O and Caki-1 cells after treatment with Tat-HSPE1. **(F)** Multiple sequence alignment of HSPE1 of different mammals. The alignment was performed using the ClustalW2 and is displayed with ESPript 3.0 (https://espript.ibcp.fr/ESPript/cgi-bin/ESPript.cgi/). Identical residues are highlighted by the dark red background and conserved residues are indicated by red font. Sequence and position of the peptide were labeled by braces. **(G)** Schematic of AlphaFold 2-structure simulation of Tat-HSPE1 (https://potoyangroup.github.io/Seq2Ensemble/) and the structure was visualized by PyMOL. **(H–J)** CCK-8 assay-based cell viability of **(H)** RCC cells (A498, 786-O and Caki-1), **(I)** normal epithelial cells (HKC, THLE-3 and HUVEC) and **(J)** other cancer cell lines (AGS, HeLa and PLC/PRF/5) treated with gradient concentrations of Tat-HSPE1 for 24 h. Data are presented as mean ± SD, n = 3. *p < 0.05, **p < 0.01, ***p < 0.001, ns, not significant.

Based on the same dataset (Supplementary Table S1), we selected a peptide originating from the heat shock protein HSPE1, specifically choosing the shorter fragment with a more significant p-value. In parallel, three peptides derived from distinct precursor proteins were randomly selected as preliminary controls. Then we designed cell-permeable fusion peptides composed of the cell-penetrating peptide Tat (Maani et al., 2024) attached to the N-terminal of these peptides. We further characterized their physicochemical properties and found that all four peptides are cationic and exhibit favorable aqueous solubility (Figure 1A). To confirm if the viability of ccRCC cells was affected by these peptides, we conducted the cell viability xperiments in which 786-O and Caki-1 cell lines were exposed to 100 μg/mL of peptides for 24 h, 48 h, and 72 h. We found that Tat-HSPE1 markedly suppressed the viability of renal carcinoma cells compared to the other peptides (Figures 1B,C). In addition, colony forming assays on 786-O and Caki-1 cells demonstrated that the proliferation capacity of ccRCC was significantly inhibited by Tat-HSPE1 in a dose-dependent manner (Figures 1D,E).

To further characterize HSPE1, *hspe1* amino acid sequences across multiple species were aligned and the location of the core peptide segment within the precursor protein was annotated. Comparative analysis revealed that this peptide region exhibited a notable degree of conservation among mammals (Figure 1F). Furthermore, AlphaFold 2-predicted secondary structures demonstrated that Tat-HSPE1 formed an α-helix (Figure 1G). Then, we tested the bioactive function of Tat-HSPE1 on different human RCC cell lines, A498, 786-O and Caki-1, which were exposed to different concentrations of Tat-HSPE1 over 24 h. A CCK-8 assay showed that, when compared to saline control, the viability of the renal cancer cell lines was significantly reduced by Tat-HSPE1 in a dose-dependent manner (Figure 1H). Notably, Tat-HSPE1 showed little effect on human renal tubular epithelial cells (HKC), normal liver cells (THLE-3) and human umbilical vein endothelial cells, HUVEC (Figure 1I). Furthermore, Tat-HSPE1 had almost no effect on AGS gastric cancer cells, cervical cancer cells (HeLa) and hepatoma cells (PLC/PRF/5) (Figure 1J). Taken together, Tat-HSPE1 inhibited the proliferation of ccRCC in a dose-dependent manner and was less cytotoxic to normal cells and other cancer cells. These results suggest that Tat-HSPE1 is to some extent, safe and cell-specific for the treatment of ccRCC.

### Tat-HSPE1 restrains the malignant progression of ccRCC

**FIGURE 2:**
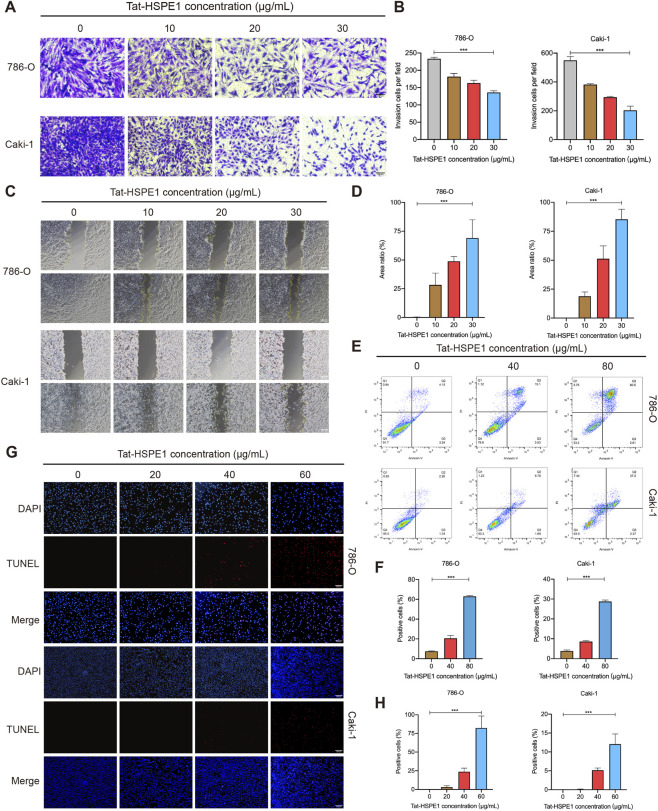
Tat-HSPE1nhibited the migration and invasion as well as induced DNA damage and apoptosis of ccRCC. **(A,B)** Cells were treated with Tat-HSPE1 (0, 10, 20, 30 μg/mL) and transwell assays show that Tat-HSPE1 inhibits the invasion of 786-O and Caki-1 cells. **(C,D)** Following treatment with Tat-HSPE1 (0, 10, 20, 30 μg/mL), wound healing assays show that Tat-HSPE1 inhibited the migration of 786-O and Caki-1 cells. **(E,F)** Cells treated with Tat-HSPE1 (0, 40, 80 μg/mL) were subjected to flow cytometric assays and the data show that Tat-HSPE1 induces apoptosis of 786-O and Caki-1 cells. **(G,H)** TUNEL assays on cells treated with Tat-HSPE1 (0, 20, 40, 60 μg/mL) show that Tat-HSPE1 induced DNA damage in 786-O and Caki-1 cells. Data are presented as mean ± SD, n = 3. *p < 0.05, **p < 0.01, ***p < 0.001, ns, not significant.

To define the functional role of Tat-HSPE1 in RCC, we performed experimental investigations across multiple features. Transwell assays revealed that Tat-HSPE1 markedly inhibited the invasion potential of ccRCC cells (Figures 2A,B). Furthermore, after exposure to different concentrations of Tat-HSPE1, ranging from 10 to 30 μg/mL, ccRCC cell migration velocity was reduced compared with the control group (Figures 2C,D).

During the initial screening, ccRCC cell exposure to high concentrations of Tat-HSPE1 resulted in extensive cell death, as observed under light microscopy. Quantitative analysis by flow cytometry revealed that Tat-HSPE1 compromised the plasma membrane integrity of 786-O and Caki-1 cells, leading to cell death (Figures 2E,F). Notably, the morphological features of Tat-HSPE1–induced cell death under bright-field microscopy differed from those induced by staurosporine (Supplementary Figure S1A), a classical apoptosis inducer (Iosub-Amir et al., 2019). We further examined classical apoptosis-related markers but did not detect any active cleaved forms of apoptosis-associated proteins (Supplementary Figure S1B), suggesting that Tat-HSPE1 induces cell death through a non-apoptotic mechanism. TUNEL assays revealed a marked dose-dependent increase in positive cells following Tat-HSPE1 treatment, indicating that Tat-HSPE1 induced genomic DNA fragmentation in ccRCC cells (Figures 2G,H). H2A.X, a member of the histone H2A family, undergoes rapid phosphorylation by the master kinase ATM (Ataxia-Telangiectasia Mutated) or PRKDC (DNA-dependent protein kinase catalytic subunit) in response to DNA double-strand breaks (DSBs), giving rise to γH2A.X—a well-established DBS marker (Menjivar et al., 2025; Chen O. et al., 2025). Immunofluorescence analyses demonstrated that Tat-HSPE1 triggered γH2A.X foci formation (Supplementary Figures S2A,B), and western blot analyses confirmed increased levels of γH2A.X in ccRCC cells after Tat-HSPE1 treatment (Supplementary Figures S2C,D), substantiating its role in inducing DNA damage. These *in vitro* findings collectively suggest that Tat-HSPE1 could impede the progression of ccRCC.

### Tat-HSPE1 triggers lysosomal membrane permeabilization (LMP) in ccRCC

**FIGURE 3:**
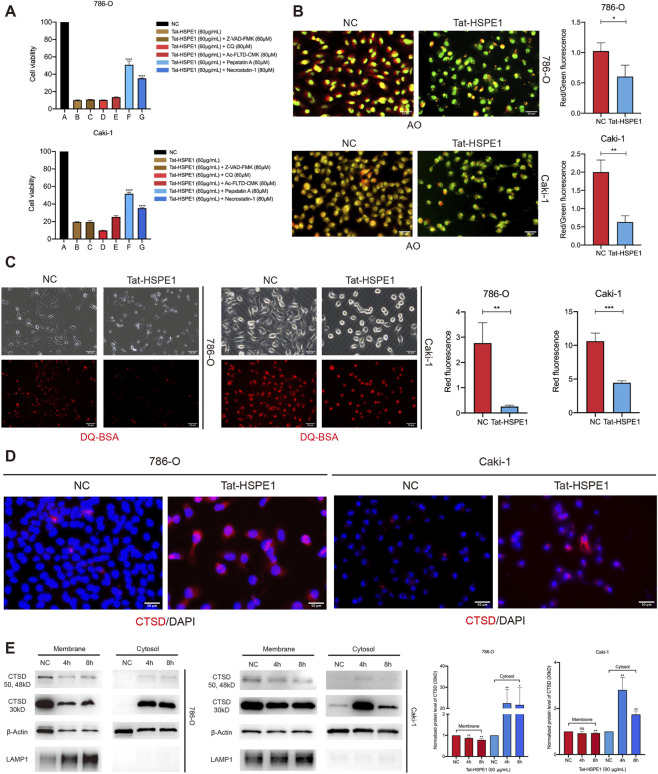
Tat-HSPE1 impairs lysosomal integrity in ccRCC cells. **(A)** CCK-8 assay measuring cell viability of 786-O and Caki-1 cells treated with Tat-HSPE1 for 24 h following pretreatment with Z-VAD-FMK, CQ, Ac-FLTD-CMK, Pepstatin A and Necrostatin-1 for 1 h. **(B)** Acridine orange (AO) staining detecting lysosome integrity in untreated and Tat-HSPE1–treated 786-O and Caki-1 cells. **(C)** DQ-BSA fluorescence (red) staining detecting lysosomal function in untreated and Tat-HSPE1–treated 786-O and Caki-1 cells. **(D)** IF assay showing subcellular localization of Cathepsin D in untreated and Tat-HSPE1–treated 786-O and Caki-1 cells. **(E)** Western blot detection of Cathepsin D levels in the cytosol and membrane fractions of 786-O and Caki-1 cells treated with Tat-HSPE1. β-Actin expression represents the loading control. LAMP1 is shown as cell compartment control (membrane fraction). Data are presented as mean ± SD. *p < 0.05, **p < 0.01, ***p < 0.001, ns, not significant.

We have demonstrated that Tat-HSPE1 induced DNA damage and triggered non-apoptotic cell death. To further elucidate the underlying mechanism of Tat-HSPE1–induced cytotoxicity, a panel of cell death inhibitors, including Z-VAD-FMK (pan-caspase inhibitor) (Zhu et al., 2007), CQ (autophagy inhibitor) (Jin et al., 2025), Ac-FLTD-CMK (pyroptosis inhibitor) (Yang et al., 2018), pepstatin A (aspartic protease and cathepsin D, E inhibitor) (Zhang et al., 2022) and necrostatin-1 (RIPK1 and cathepsin D inhibitor) (Zhang et al., 2025a; Jantas et al., 2020), were tested. 786-O and Caki-1 cells were pretreated with each inhibitor prior to Tat-HSPE1 administration. Notably, pepstatin A and Necrostatin-1 partially rescued cells from Tat-HSPE1–induced death (Figure 3A), implicating the involvement of Cathepsin D in Tat-HSPE1–induced cytotoxicity. These results proposed that Tat-HSPE1 induced lysosome-dependent cell death. Lysosome-dependent cell death is demarcated by lysosomal membrane permeabilization (LMP), resulting in the release of lysosomal contents, mainly the cathepsin family, into the cytoplasm (Galluzzi et al., 2018).

To determine whether Tat-HSPE1 induced LMP in ccRCC cells, we first performed acridine orange (AO) staining on untreated or Tat-HSPE1–treated 786-O and Caki-1 cells. AO is a lysosomotropic metachromatic dye that fluoresces red within intact lysosomes but shifts to green upon redistribution into the cytosol following lysosomal destabilization (Shao et al., 2025; Wang et al., 2024). Notably, Tat-HSPE1 treatment led to a marked reduction in red fluorescence, indicative of LMP (Figure 3B). Lysosomes are the terminal end of the endocytic pathway. To further assess lysosomal integrity, DQ-BSA (Dye-Quenched Bovine Serum Albumin), a self-quenched fluorescent substrate of lysosomal proteases that emits bright red fluorescence upon proteolytic cleavage within functional lysosomes, was used (Xu et al., 2022). A significant decrease in DQ-BSA–derived fluorescence intensity was observed in Tat-HSPE1–treated cells, suggesting that Tat-HSPE1 impairs lysosomal integrity in ccRCC cells (Figure 3C). The release of cathepsin D from the lysosome lumen into the cytosol in ccRCC cells after treatment with Tat-HSPE1 further supported the role of Tat-HSPE1 in inducing LMP (Figure 3D). Cathepsin D exists in three principal molecular forms of approximately 50, 48 and 30 kDa. The 50 kDa species represents the zymogen (pro-cathepsin D), the 48 kDa form corresponds to an intermediate processing product, and the 30 kDa species constitutes the mature, enzymatically active form. Upon LMP, the mature 30 kDa cathepsin D will be released into the cytosol (Khalkhali-Ellis and Hendrix, 2014). Tat-HSPE1–treated or non-treated 786-O and Caki-1 cells were separated into cytosolic and membrane fractions, and consistent with the immunofluorescence analysis, western blot visualization revealed a marked accumulation of the mature 30 kDa species within the cytoplasmic fraction following Tat-HSPE1 treatment (Figure 3E). Taken together, our results demonstrate that LMP occurs in Tat-HSPE1–treated ccRCC cells, leading to plasma membrane rupture and the release of lysosomal contents.

### Tat-HSPE1 promotes autophagic flux and modulates autophagy in ccRCC

**FIGURE 4:**
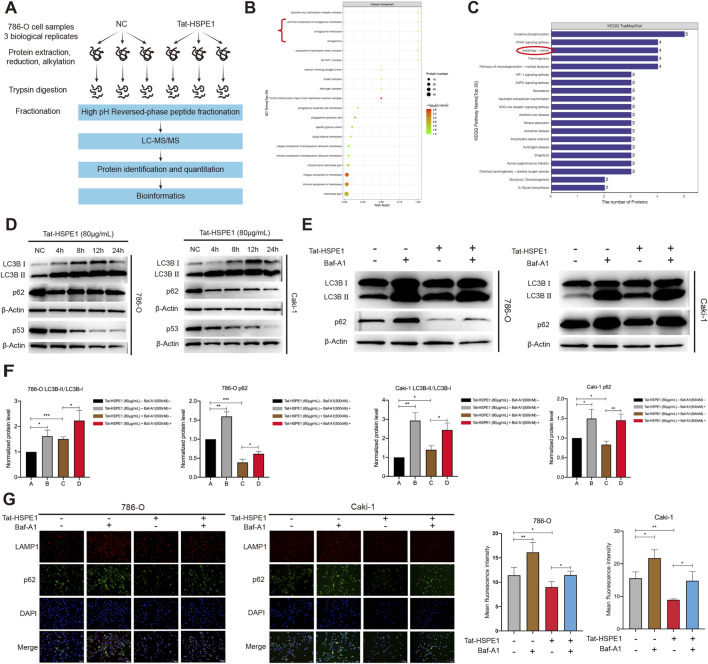
Tat-HSPE1 promotes autophagic flux in ccRCC. **(A)** Flowchart of 4D-label-free quantitative proteome and bioinformatics analysis in Tat-HSPE1–treated and untreated 786-O cells. **(B)** Top 20 Cellular Component enrichment analysis of the GO annotation in 786-O cells treated with Tat-HSPE1. **(C)** KEGG pathway enrichment analysis to identify the top 20 canonical pathways in 786-O cells treated with Tat-HSPE1. **(D)** Western blot analysis detecting the levels of autophagy-related proteins (LC3B-I, LC3B-II, p62, p53) of Tat-HSPE1–treated (4, 8, 12 and 24 h) and untreated 786-O and Caki-1 cells. Protein levels of β-Actin were detected as a loading control. **(E,F)** Western blot readouts of the expression of LC3B and p62 proteins in Tat-HSPE1–treated and untreated 786-O and Caki-1 cells with or without Baf-A1. β-Actin expression was detected as a loading control. **(G)** Immunofluorescence analysis detecting the mean fluorescence intensity of endogenous p62 puncta in Tat-HSPE1–treated 786-O and Caki-1 cells with or without Baf-A1 for 4 h. Data are presented as mean ± SD. *p < 0.05, **p < 0.01, ***p < 0.001, ns, not significant.

To investigate the potential molecular mechanism of Tat-HSPE1 cytotoxicity, we determined the proteome profile of the group of cells treated with 60 μg/mL Tat-HSPE1 and compared it to the control group (Figure 4A). Approximately 6,000 proteins were detected collectively in the treatment group and the control group (Supplementary Figure S3A). A total of 96 proteins exhibited statistically significant changes in expression in the Tat-HSPE1–treated group compared to that in the control group, with the expression of 51 proteins increased more than 2.0-fold, and 45 proteins exhibited expression levels at less than 0.5-fold of the controls (Supplementary Figures S3B, S3E; Supplementary Table S2). Among these differentially expressed proteins, we noted a few molecules involved in cell death pathways such as pyroptosis (GSDMD) (Shi et al., 2015), necroptosis (TNFRSF10A) (Kaczynski et al., 2023), and autophagy (CD63) (Waisner et al., 2023) (Supplementary Figure S3C), consistent with the morphological alterations observed in ccRCC cells following Tat-HSPE1 treatment. Ourobservation further validated the earlier findings that Tat-HSPE1 induces cell death, suggesting that, as an exogenous stimulus, Tat-HSPE1 may engage complex and multifaceted cell death pathways.

As shown in Supplementary Table S3, expression of 31 proteins was observed in at least two or three replicates in one group, while the other group showed no recorded values, with 21 proteins detected in the Tat-HSPE1–treated group and 10 proteins detected in control groups. Organelles are membrane-bound subcellular compartments with distinct structures and functions, serving as critical platforms for protein activity and cellular processes (Netzer et al., 2023). Subcellular localization analysis revealed that the differentially expressed proteins localized predominantly to the nucleus and cytoplasm (Supplementary Figure S3D). To investigate the latent function of the differentially expressed proteins, Gene Ontology (GO) functional annotation was performed. Cellular component enrichment analysis showed that proteins in the “extrinsic component of omegasome membrane”, “omegasome membrane” and “omegasome” sets were significantly enriched in the Tat-HSPE1–treated group compared to that in the control group (Figure 4B). Omegasome is a dynamic membrane structure originating from the endoplasmic reticulum that plays a pivotal role in the early stages of autophagosome biogenesis (Norell et al., 2024; Roberts and Ktistakis, 2013). The omegasome is a structural and functional hub for the initiation of autophagy, rendering it indispensable for autophagosome formation. This was consistent with the KEGG pathway analysis which identified autophagy as one of the top five enriched pathways (Figure 4C). As Tat-HSPE1 was previously shown to induce lysosomal membrane permeabilization—a process tightly linked to autophagy—we postulated that Tat-HSPE1 may promote autophagy in ccRCC cells.

To further demonstrate the role of autophagy in Tat-HSPE1–induced cell death, we treated 786-O and Caki-1 cells with 80 μg/mL of Tat-HSPE1 and assessed the expression of key autophagy-related proteins at multiple time points (Figure 4D). A significant increase in LC3B-II accumulation was observed in the treated cells. During autophagy, LC3B (microtubule-associated protein 1 light chain 3B) is initially cleaved by Atg4 protease to generate cytosolic LC3B-I, which is modified and processed by the ubiquitin-like conjugation system involving Atg7 and Atg3, to produce LC3B-II. The abundance of LC3B-II correlates positively with the degree of autophagy in cells (De Mazière et al., 2022). We further examined the levels of p62 (also known as SQSTM1, Sequestosome-1), a selective autophagy substrate whose accumulation inversely correlates with autophagic flux (Zein et al., 2025). Notably, Tat-HSPE1 treatment led to a marked reduction in p62 levels, indicating enhanced autophagic activity. Additionally, we observed decreased expression of p53 following Tat-HSPE1 treatment. It has been previously established that several autophagy inducers, such as rapamycin and tunicamycin, promote the rapid degradation of p53 (Tasdemir et al., 2008).

Bafilomycin A1 (Baf-A1) blocks autophagosome-lysosome fusion and inhibits autophagy at a late stage (Ahmad et al., 2025). LC3 lipidation was represented as LC3-II/LC3-I, which positively correlates with autophagic flux. Co-treatment with Baf-A1 will increase the LC3-II to LC3-I ratio once autophagy is induced. Thus, we used western blots to examine for changes in autophagic flux by detecting the conversion of LC3B-I to LC3B-II and the expression of p62. 786-O and Caki-1 cells were treated with Tat-HSPE1 for 4 h in the presence or absence of Baf-A1. Tat-HSPE1+Baf-A1 exposure markedly decreased p62 protein levels and increased the LC3B-II/LC3B-I ratio, indicating enhanced autophagic activity (Figures 4E,F). To test the possibility that Tat-HSPE1 enhances autophagic flux, we also performed an immunofluorescence assay using anti-p62 antibody to quantify endogenous p62. Consistent with the western blot readouts, we observed that the endogenous p62 puncta signal diminished after treatment with Tat-HSPE1, but p62 levels were rescued by Baf-A1 which blocked autophagic flux (Figure 4G). Hence, our data collectively demonstrate that autophagy is indeed induced by Tat-HSPE1 in ccRCC cells.

### Tat-HSPE1 interacts with CTTNBP2NL, a negative regulator of autophagy

To follow up on the confirmation that Tat-HSPE1 induced autophagy in ccRCC cells, we sought to identify the unknown target protein of Tat-HSPE1. A tag-conjugated peptide, Biotin-PEG3-Tat-HSPE1, was synthesized and used to evaluate its antitumor efficacy and functional integrity in 786-O cells. CCK-8 analysis revealed that both Biotin-PEG3-Tat-HSPE1 and Tat-HSPE1 exhibited comparable inhibitory effects on cell viability (Supplementary Figure S4A), suggesting that the addition of the synthetic tag did not compromise the biological activity of Tat-HSPE1. We next examined the subcellular localization of Biotin-PEG3-Tat-HSPE1 using immunofluorescence microscopy. Preliminary fluorescence analysis indicated that Biotin-PEG3-Tat-HSPE1 was predominantly localized to the nucleolus (Supplementary Figure S4B). Fibrillarin (FBL) is a canonical nucleolar protein that plays a critical role in ribosome biogenesis by promoting rDNA transcription and maintaining the structural integrity of the fibrillar center (Zhang et al., 2026). Taking advantage of this property, we used an anti-FBL antibody to label nucleoli. Co-immunostaining revealed a strong colocalization of Biotin-PEG3-Tat-HSPE1 with FBL (Supplementary Figure S4C), indicating that, upon cellular entry, Tat-HSPE1 primarily accumulates in the nucleolar compartment.

**FIGURE 5:**
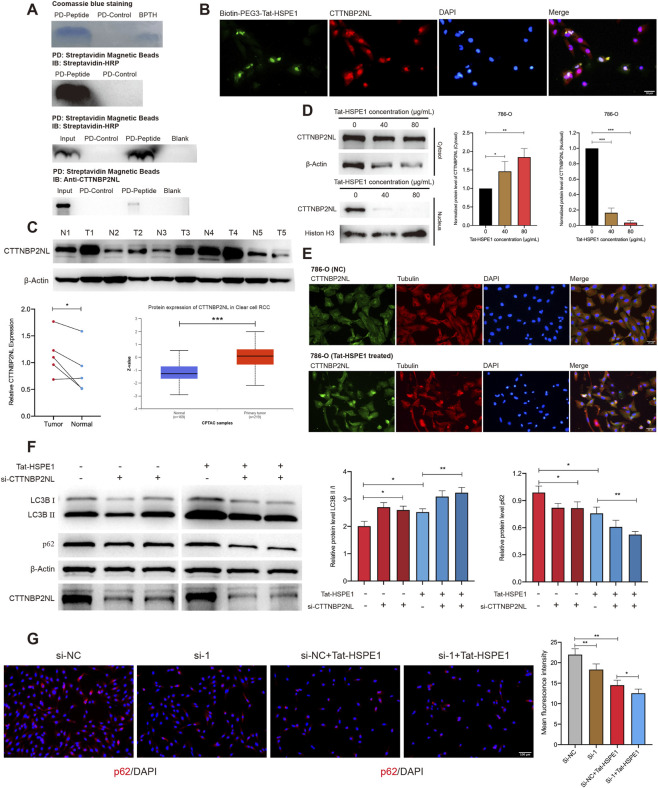
Tat-HSPE1 interacts with CTTNBP2NL and promotes autophagy. **(A)** Coomassie blue staining and western blot of the eluted proteins from affinity columns, BPTH: Biotin-PEG3-Tat-HSPE1; western blot analysis of the eluted proteins from the biotin pull-down assay using an Anti-CTTNBP2NL antibody and streptavidin-HRP. **(B)** Immunofluorescence assay showing localization of Biotin-PEG3-Tat-HSPE1 and CTTNBP2NL in 786-O cells. **(C)** CTTNBP2NL protein levels in 5 pairs of ccRCC tissues and controls were assessed by western blotting. β-Actin expression was detected as a loading control. Differential expression of CTTNBP2NL protein between ccRCC and normal tissues in CPTAC samples. **(D)** Western blot analysis detecting levels of CTTNBP2NL in cytosolic and nuclear fractions of Tat-HSPE1–treated and untreated 786-O cells. β-Actin and Histon H3 expression were detected as a loading control. **(E)** IF assay showing subcellular localization change of CTTNBP2NL and Tubulin in untreated and Tat-HSPE1–treated 786-O cells. **(F)** Western blot analysis detecting the levels of LC3B and p62 in CTTNBP2NL siRNA (*si-NC*, *si-1*, *si-2*) transfected 786-O cells with or without Tat-HSPE1 treatment. β-Actin expression was detected as a loading control. **(G)** IF analysis detecting the mean fluorescence intensity of endogenous p62 puncta in CTTNBP2NL siRNA (*si-NC*, *si-1*) transfected 786-O cells with or without Tat-HSPE1 treatment. n = 3. Data are presented as mean ± SD. *p < 0.05, **p < 0.01, ***p < 0.001, ns, not significant.

To further clarify the molecular interactions involving Tat-HSPE1, we performed pull-down assays using streptavidin-conjugated magnetic beads to capture Biotin-PEG3-Tat-HSPE1. Following affinity purification, SDS-PAGE confirmed successful retrieval of the peptide, and the entire content of the gel lanes (Biotin-PEG3-Tat-HSPE1, Biotin-PEG3-NH2) was subsequently subjected to liquid chromatography–tandem mass spectrometry (LC-MS/MS) to identify interacting proteins. Among the top 20 most abundant proteins identified by LC-MS/MS (Supplementary Table S4), CTTNBP2NL exhibited the most similar subcellular localization pattern to Tat-HSPE1. Subsequent validation by western blot confirmed that endogenous CTTNBP2NL was co-immunoprecipitated with Biotin-PEG3-Tat-HSPE1 (Figure 5A). Furthermore, immunofluorescence analysis demonstrated robust colocalization of CTTNBP2NL and Biotin-PEG3-Tat-HSPE1 (Figure 5B), supporting CTTNBP2NL as an interacting partner of Tat-HSPE1. We assessed CTTNBP2NL expression in five paired samples of ccRCC and adjacent normal tissues. CTTNBP2NL was upregulated in ccRCC tissues, consistent with the results in the CPTAC (Clinical Proteomic Tumor Analysis Consortium) database (https://hupo.org/Clinical-Proteome-Tumor-Analysis-Consortium-(CPTAC) (Figure 5C), and similar results were noted for ccRCC patients of different stages (Supplementary Figure S5A), ages (Supplementary Figure S5B), weights (Supplementary Figure S5C) and sexes (Supplementary Figure S5D), implicating a potential pro-tumorigenic role for this protein in renal cancer. Furthermore, CTTNBP2NL gene expression in ccRCC was negatively correlated with that of HSPE1 (Supplementary Figure S5E).

Next, cells were treated with increasing concentrations of Tat-HSPE1 to assess the potential effect of Tat-HSPE1 on CTTNBP2NL. After the indicated incubation, nuclear and cytoplasmic fractions were isolated as CTTNBP2NL localizes to both compartments. Isolation was based on the Nuclear and Cytoplasmic Protein Extraction Kit, and protein levels were assessed by western blots. Tat-HSPE1 treatment significantly decreased nuclear CTTNBP2NL while increasing its cytoplasmic abundance when compared with controls (Figure 5D), indicating peptide-induced nuclear-to-cytoplasmic translocation. Immunofluorescence analysis corroborated these findings, revealing cytoplasmic enrichment of CTTNBP2NL following peptide exposure. Additionally, Tat-HSPE1 induced nuclear shrinkage and severe cytoskeletal disruption, characterized by microtubule disorganization (Figure 5E). Together, these data demonstrate that Tat-HSPE1 redistributes CTTNBP2NL and compromises cytoskeletal integrity.

Different databases were used to perform enrichment analysis of CTTNBP2NL-related pathways. The analysis indicated that CTTNBP2NL was predominantly enriched in the hippo signaling pathway, a pathway that modulates cell proliferation, differentiation, and survival (Supplementary Figures S5F, G). CTTNBP2NL is a component of the STRIPAK (striatin-interacting phosphatase and kinase) complex, which plays an important role in the hippo signaling pathway. Studies have shown complex interactions between the hippo signaling and autophagic pathways. CTTNBP2NL may participate in regulating the autophagic pathway through YAP (Yes-associated protein) signaling or the MAP4K-family (Seo et al., 2024; Zhou et al., 2024). To evaluate the function of CTTNBP2NL in autophagy, we treated 786-O cells with negative control small interfering RNA (*si-NC*) and siRNAs directed against CTTNBP2NL (*si-1*, *si-2*). Knockdown of CTTNBP2NL led to a reduction in p62 levels and an elevation in LC3B-II/LC3B-I,both of which intensified upon treatment with Tat-HSPE1 (Figure 5F). Consistently, endogenous p62 puncta signal diminished after knockdown of CTTNBP2NL in combination with Tat-HSPE1, which contributed to a synergistic downstream effect on autophagy regulation (Figure 5G). Collectively, our data indicate that CTTNBP2NL is a Tat-HSPE1-interacting protein that negatively regulates autophagy.

### Tat-HSPE1 effectively inhibits tumor growth *in vivo*

**FIGURE 6:**
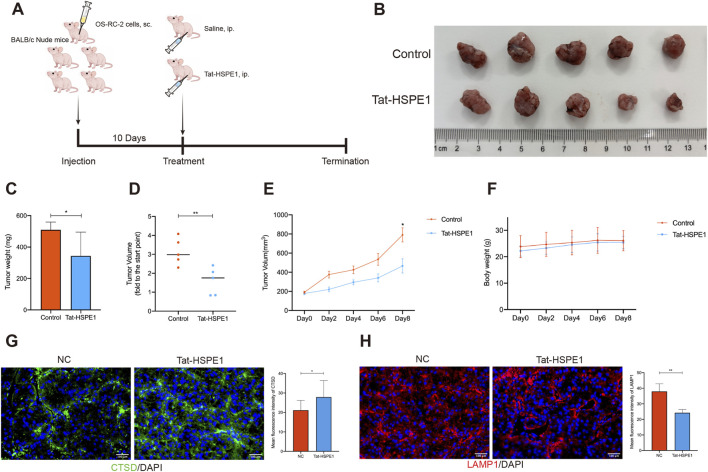
Tat-HSPE1 effectively inhibits tumor growth *in vivo*. **(A)** Schematic overview of treatment with Tat-HSPE1 in xenograft nude mouse models. i. p., intraperitoneal. **(B)** Tumor images in control and Tat-HSPE1–treated groups. **(C,D)** Tumors were weighted **(C)** and the tumor growth ratio **(D)** was calculated once the mice were euthanized. **(E)** Tumor volumes were monitored every other day. **(F)** Mice body weight over the duration of the experiment. **(G)** Immunofluorescence analysis of Cathepsin D in tumor tissues and the mean fluorescence intensity of each group. **(H)** Immunofluorescence analysis of LAMP1 in tumor tissues and the mean fluorescence intensity of each group. n = 3. Data are presented as mean ± SEM **(E)** or mean ± SD **(C,D,G,H)**. *p < 0.05, **p < 0.01, ***p < 0.001, ns, not significant.

Subcutaneous xenograft models in nude mice were developed to investigate the *in vivo* antitumor efficacy of Tat-HSPE1. Approximately 2 × 10^6^ OS-RC-2 human RCC cells were injected subcutaneously into the flanks of the mice. Once tumor volumes reached approximately 200 mm^3^, the mice were randomized into treatment cohorts and administered Tat-HSPE1 (30 mg/kg) or an equivalent volume of saline via intraperitoneal injection once daily (Figure 6A). During the experiment, tumor growth was monitored every other day by caliper measurements. The tumors of Tat-HSPE1 treated mice were smaller than those of the control group (Figure 6B). At the conclusion of the observation period, excised tumors from the Tat-HSPE1–treated mice displayed reduced weights and tumor growth ratios relative to the controls (Figures 6C,D). Tat-HSPE1 treatment resulted in a significant reduction in tumor volume compared to the control group (Figure 6E). Furthermore, Tat-HSPE1 was also well tolerated in mice based on their stable body weight (Figure 6F).

To verify the antitumor mechanisms of Tat-HSPE1, we analyzed the expression of LMP-related markers in tumor tissues. Immunofluorescence analysis revealed that Cathepsin D (CTSD), a key lysosomal protease, exhibited increased expression and a greater diffuse cytoplasmic distribution in the treatment group (Figure 6G), consistent with LMP activation. Moreover, the fluorescence intensity of LAMP1 in Tat-HSPE1 treatment groups was significantly weakened, indicating that Tat-HSPE1 compromised the lysosomal membrane of cancer cells (Figure 6H). These findings align with our previous *in vitro* observations and suggest that Tat-HSPE1 suppresses RCC tumor growth by promoting autophagy and inducing LMP. Taken together, these results demonstrate that Tat-HSPE1 exerts potent antitumor effects *in vivo* and may have potential as a molecular tool in the treatment of RCC.

## Materials and methods

### Sample collection

Paired clear cell renal cell carcinoma (ccRCC) tissues and adjacent non-tumorous tissues were obtained from three patients at Tongren Hospital, Shanghai Jiao Tong University School of Medicine. Written informed consent was obtained from all participants prior to sample collection. The study was approved by the Institutional Ethics Committee of Tongren Hospital. None of the patients received local or systemic therapy before surgery. Immediately after surgical resection, tissue specimens were frozen in liquid nitrogen and stored for subsequent peptidomic analysis. Additional paired samples from five patients were collected under identical conditions for western blot analysis.

### Peptide identification, bioinformatics analysis and peptide synthesis

Full methodological details are described in *Tat-hspb1 Suppresses Clear Cell Renal Cell Carcinoma (ccRCC) Growth via Lysosomal Membrane Permeabilization* (Zhang et al., 2022), Peptide Tat-HSPE1, Tat-PPP2R1A, Tat-PKM, Tat-PGK1, Biotin-PEG3-Tat-HSPE1 were synthesized by Science Peptide Biological Technology (Shanghai, China) through the solid-phase method and dissolved in double-distilled water.

### Cell culture

Human RCC cell lines (786-O, A498, and Caki-1), normal human liver cell lines (THLE-3), human gastric epithelial cell lines (AGS), and human umbilical vein endothelial cells (HUVEC) were obtained from the Shanghai Institute of Cell Biology, Chinese Academy of Sciences. Human renal tubular epithelial cells (HKC) and hepatoma cells (PLC/PRF/5) were purchased from Procell (Wuhan, China). 786-O, Caki-1, HUVEC and THLE-3 cells were cultured in RPMI-1640 medium (Gibco); HKC cells were cultured in DMEM/F12 medium (Procell); A498, AGS, HeLa and PLC/PRF/5 cells were cultured in DMEM medium (Gibco). Mediums were supplemented with 10% fetal bovine serum (Gibco) and 1% P/S (Gibco). All cells were mycoplasma-free and maintained at 37 °C in a humidified atmosphere with 5% CO2.

### Cell viability assay

Cells were seeded in 96-well plates at a density of 3 × 10^3^ cells per well. After treatment with the indicated agents for specified durations, Cell Counting Kit-8 (CCK-8; TargetMol) reagent was added and incubated for 2 h. Absorbance at 450 nm was measured using a Multiskan FC Microplate Reader (Thermo Scientific).

### Wound healing assay

Caki-1 and 786-O cells were seeded in 12-well plates at a density of 5 × 10^5^ cells per well. After 48 h, a scratche was introduced into the cell monolayer. Detached cells were removed with PBS, and cells were cultured in RPMI-1640 containing 1% FBS and Tat-HSPE1 (0, 10, 20, and 30 μg/mL). Wound closure was monitored at indicated time points using an inverted microscope (Nikon), and migration rates were quantified using ImageJ software.

#### Colony formation assay

786-O and Caki-1 cells were seeded in 6-well plates at a density of 2 × 10^3^ cells per well and treated with Tat-HSPE1 (0, 10, 20, and 30 μg/mL). After 7 days, and colonies were fixed in methanol for 20 min at room temperature, stained with 0.2% crystal violet in methanol for 15 min, and imaged. Colony numbers were quantified using ImageJ.

### TUNEL assay

TUNEL staining was performed using a commercial kit (Beyotime) according to the manufacturer’s protocol. Cells (1 × 10^5^ per well) were seeded in 24-well plates and treated with Tat-HSPE1 at indicated concentrations for 24 h. Cells were fixed in methanol for 20 min at room temperature, permeabilized with 0.5% Triton X-100, and incubated with the TUNEL reaction mixture at 37 °C for 1 h. Nuclei were counterstained with DAPI (Beyotime) and imaged using a TS2-FL fluorescence microscope (Nikon). The percentage of TUNEL-positive cells was quantified relative to the total nuclei.

#### Transwell cell invasion assays

Transwell invasion assays were performed using Matrigel-coated 24-well chambers (Corning). Cells (2 × 10^4^ per well) suspended in serum-free medium were seeded into the upper chamber, while medium containing 10% FBS was added to the lower chamber. After 48 h of incubation, non-invading cells on the upper surface were removed, and invaded cells were fixed in methanol and stained with 0.2% crystal violet. Cells were imaged and quantified under a microscope.

### Flow cytometry analysis

Apoptosis was assessed using Annexin V-FITC/PI staining followed by flow cytometry. Briefly, cells were treated with Tat-HSPE1 (0, 40, and 80 μg/mL) for 24 h. Cells were harvested and stained with FITC–Annexin V and PI (Absin) for 15 min at room temperature in the dark. Data were acquired using FACS auto flow cytometer (BD Biosciences) and analyzed with FlowJo software.

#### AO staining and DQ-BSA assay

786-O and Caki-1 cells (4 × 10^5^ per well) were seeded in 12-well plates and treated with Tat-HSPE1 (80 μg/mL) for 2 h at 37 °C. Cells were then stained with acridine orange (50 μg/mL; Sigma-Aldrich) in complete medium for 15 min and imaged using a fluorescence microscope (Nikon). Red and green fluorescence intensities were analyzed using ImageJ software.

For DQ-BSA assay, cells (8 × 10^5^ per well) were seeded in 6-well plates and treated with Tat-HSPE1 (80 μg/mL) for 4 h. After that, cells were incubated with DQ-BSA (12.5 μg/mL; Share-bio) for an additional 8 h. Both bright-field and fluorescence images were acquired using a fluorescence microscope. Fluorescence intensity was analyzed using ImageJ software.

### Fractionation of cytosol and membrane extracts

Cells were washed with ice-cold PBS and resuspended in cytosol extraction buffer (250 mM sucrose, 10 mM KCl, 1.5 mM MgCl_2_, 1 mM EDTA, 1 mM EGTA, 20 mM HEPES) supplemented with 250 μg/mL digitonin, followed by incubation on ice for 10 min. Lysates were centrifuged at 13,000 × g for 90 s, and the resulting supernatant (cytosolic fraction) was collected. Pellets (membrane fraction) were resuspended in lysis buffer and centrifuged at 13,000 × g for 15 min at 4 °C, and the supernatant was collected as the membrane extract (Min and Kwon, 2020).

### siRNA suppression of gene expression

siRNAs targeting human CTTNBP2NL or negative control were synthesized and purified by HANBIO (Shanghai, China). The sequences of siRNAs used in this study are listed in Supplementary Table S5 siRNAs were transfected into 786-O cells using RNA transfection reagent (RNAFit; HANBIO) according to the manufacturer’s advising protocol. Knockdown efficiency was confirmed by western blot 48–72 h post-transfection.

### Immunofluorescence

786-O and Caki-1 cells were inoculated on coverslips and incubated overnight. Cells were treated with different agents under different experimental conditions (Tat-HSPE1, staurosporine, Baf-A1, SiRNA). After treatment, cells were fixed in ice-cold methanol for 20 min, permeabilized with 0.5% Triton X- 100 solution for 10 min and blocked with 1% bovine serum albumin (BSA) for 30 min. Cells were incubated with primary antibodies overnight at 4 °C, followed by incubation with fluorescent secondary antibodies (Proteintech) for 1 h at room temperature. Nuclei were stained with DAPI, and images were acquired using a fluorescence microscope (Nikon).

Xenograft tumor tissues were fixed in 4% paraformaldehyde and then dehydrated and embedded in paraffin. Embedded tissue was performed in 3 μm thick sections followed by deparaffinization and antigen retrieval. Tissue sections were treated with 3% H_2_O_2_ to quench endogenous peroxidase activity and incubated with 5% BSA to block non-specific binding. In brief, sections were incubated with primary antibodies and fluorescent secondary antibodies and visualized under fluorescence microscope.

For biotin-labeled peptide detection, cells were inoculated on coverslips and treated with Biotin-PEG3-Tat-HSPE1, Biotin-PEG3-NH2 serves as a negative control. After treatment, cells were fixed with methanol for 20 min, permeabilized with 0.5% Triton X- 100 and blocked with 0.5% BSA, followed by stained with 488-Streptavidin (Share-bio).

#### Western blot and biotin pull-down assay

Cells and tissues were lysed in RIPA buffer (Beyotime) supplemented with 1 mM PMSF and a protease inhibitor cocktail. Protein extracts were fractionated by SDS–PAGE (10%, 12.5%, or 15%) and transferred to PVDF membranes. Membranes were blocked with 5% non-fat milk for 1 h at room temperature and incubated overnight at 4 °C with the appropriate primary antibodies. After washing, membranes were incubated with HRP-conjugated secondary antibodies for 1 h at room temperature and visualized using enhanced chemiluminescence (Thermo Fisher).

For the biotin pull-down assay, streptavidin-coated magnetic beads (Share-bio) were incubated with Biotin-PEG3-Tat-HSPE1 and Biotin-PEG3-NH2 control respectively at 4 °C for 4 h to generate peptide–bead conjugates. 786-O cells were harvested by trypsinization and centrifugation, then lysed in buffer (20 mM HEPES, 137 mM NaCl, 1 mM EDTA, 0.2% Triton X-100, 10% glycerol, and protease inhibitors) (Shoji-Kawata et al., 2013). Cell suspensions were homogenized using a 25G syringe and cleared by high-speed centrifugation. Equal volumes of supernatants were incubated with peptide-bead complexes at 4 °C overnight. Bound proteins were eluted, separated by SDS–PAGE, visualized by Coomassie blue staining. whole gel lanes were excised and analyzed by LC-MS/MS.

### Antibodies, chemicals and commercial kits

Antibodies involved in this study: Anti-γH2A.X (Zen Bio, Cat. No. 201082-7G9), Anti-CTSD (Abcam, Cat. No. ab75852), Anti-LAMP1 (Cell Signaling Technology, Cat. No. 9091), Anti-β-Actin (Zen Bio, Cat. No. 200068-8F10), Anti-LC3B (Zen Bio, Cat. No. R381544), Anti-p62 (Abcam, Cat. No. ab207305), Anti-p53 (Santa Cruz Biotechnology, Cat. No. sc-126), Anti-PARP1 (Proteintech, Cat. No. 13371-1-AP), Anti-cleaved-PARP1 (CST, Cat. No. 5625T), Anti-caspase-3 (Proteintech, Cat. No. 19677-1-AP), Anti-caspase-8 (Proteintech, Cat. No. 13423-1-AP), Anti-caspase-9 (Proteintech, Cat. No. 10380-1-AP), Anti-α-Tubulin (Zen Bio, Cat. No. R23454), Anti-Histon H3 (Zen Bio, Cat. No. R381432), Anti-FBL (Proteintech, Cat. No. 16021-1-AP), Anti-CTTNBP2NL (Proteintech, Cat. No. 25523-1-AP), HRP conjugated Goat Anti-Mouse IgG (H + L) (Bio-Rad, Cat. No. 170–6,516), HRP conjugated Goat Anti-Rabbit IgG (H + L) (Bio-Rad, Cat. No. 170–6,515), CoraLite488 conjugated Affinipure Goat Anti-Mouse IgG(H+L) (Proteintech, Cat. No. SA00013-1), CoraLite488 conjugated Affinipure Goat Anti-Rabbit IgG(H+L) (Proteintech, Cat. No. SA00013-2), CoraLite594 conjugated Affinipure Goat Anti-Mouse IgG(H+L) (Proteintech, Cat. No. SA00013-3), CoraLite594 conjugated Affinipure Goat Anti-Rabbit IgG(H+L) (Proteintech, Cat. No. SA00013-4).

Chemicals, peptides, and recombinant proteins involved in this study: Biotin-PEG3-NH2 (AmBeed, Cat# A444152), Z-VAD-FMK (MedChemExpress, Cat# HY-16658B), Ac-TLTD-CMK (Selleckchem, Cat# S9817), Chloroquine (MedChemExpress, Cat# HY-17589A), Pepstatin A (Selleckchem, Cat# S7381), Necrostatin-1 (MedChemExpress, Cat# HY-15760), Staurosporine (Solarbio, Cat# IS04709), Baf-A1 (MeilunBio, Cat# MB5505-L), BSA (Beyotime, Cat# ST025), Acridine orange (Sigma-Aldrich, Cat# A8097), Sucrose (Sangon Biotech, Cat# A361851), Potassium chloride (Sangon Biotech, Cat# A501159), Crystal Violet Staining Solution (Beyotime, Cat# C0121), Magnesium chloride hexahydrate (Sangon Biotech, Cat# A610328), Sodium chloride (Sangon Biotech, Cat# A610476), EDTA (Beyotime, Cat# ST1305), EGTA (Beyotime, Cat# Y257087), HEPES (Beyotime, Cat# ST090), Digitonin (Beyotime, Cat# ST1272), Cell lysis buffer for Western and IP (Beyotime, Cat# P0013), Protease and phosphatase inhibitor cocktail (Beyotime, Cat# P1045), Triton X-100 (Sangon Biotech, Cat# A417820), Glycerol (Sangon Biotech, Cat# A362460), Antifade Mounting Medium with DAPI (Beyotime, Cat# P0131), DAPI (Beyotime, Cat# C1002), HRP-Streptavidin (Share-bio, Cat# SB-UE001), Streptavidin Magnetic Beads (Share-bio, Cat# SB-PR080), 488-Streptavidin (Share-bio, Cat# SB-YS0079), RNAFit (HANBIO, Cat# HB-RF-1000).

Critical commercial assay kits involved in this study: Cell Counting Kit-8 (TargetMol, Cat# C0005), One Step TUNEL Apoptosis Assay Kit (Beyotime, Cat# C1089), Nuclear and Cytoplasmic Protein Extraction Kit (Share-bio, Cat# SB-PR013HZ), Annexin V-FITC Apoptosis Assay Kit (Absin, Cat# abs50001), DQ-BSA-RED Lysozyme Assay Kit (Share-bio, Cat# D-12051SB).

### Animal experiments

Xenograft experiments were performed in accordance with the ethical guidelines and approved by the Ethics Committee of Shanghai Tongren Hospital. Male BALB/c nude mice (4 week old) were obtained from GemPharmatech (Jiangsu, China) and acclimatized in the animal laboratory center for 1 week. OS-RC-2 cells (2 × 10^6^ cells suspended in 100 μL PBS) were subcutaneously injected into the right axilla of mice. When tumors reached approximately 200 mm^3^, mice were randomly assigned to different groups for the indicated treatments. Mice were injected intraperitoneally with Tat-HSPE1 (30 mg/kg) or saline once daily, Tumor size was measured with a caliper and recorded every 2 days, the tumor volume was calculated according to the formula volume = length × width^2^ × 1/2. All mice were euthanized 10 days after treatment. Tumors were collected, weighed, and dissected to prepare paraffin-embedded tissues.

### Proteomic analysis

Cells were treated with Tat-HSPE1 (60 μg/mL) for 24 h and lysed SDT buffer (4% SDS, 100 mM Tris-HCl, 1 mM DTT, pH 7.6). Peptides generated from each sample were desalted using C18 cartridges, concentrated by vacuum centrifugation, and reconstituted in 0.1% formic acid. After FASP digestion, 20 μg of protein from each sample was separated on a 12.5% SDS-PAGE gel and subjected to LC-MS/MS analysis (Applied Protein Technology Co., Ltd., Shanghai, China). Raw MS data were integrated and processed using MaxQuant software for protein identification and quantification.

Subsequent bioinformatic analyses were performed as follows: hierarchical clustering was conducted using Cluster 3.0 and visualized with Java TreeView; subcellular localization was predicted using CELLO (http://cello.life.nctu.edu.tw/); Gene Ontology (GO) annotation was carried out with Blast2GO; and KEGG pathway annotation was performed using the Kyoto Encyclopedia of Genes and Genomes database (http://geneontology.org/).

### Statistical analysis

*In vitro* experiments were performed in at least three independent replicates. Statistical analyses were carried out using GraphPad Prism 9.0 and Microsoft Excel. Statistical significance (P-value) was assessed by unpaired or paired Student’s t-test, or one-way ANOVA, as appropriate. P < 0.05 was considered statistically significant. All data are reported as mean ± standard deviation (SD) or standard error of the mean (SEM). Software and algorithms involved in this study: GraphPad Prism 9 (GraphPad, https://www.graphpad.com), ImageJ (National Institutes of Health, https://imagej.nih.gov/ij/download.html), Endnote X9 (Clarivate, https://endnote.com), FLOWJO (BD, https://www.flowjo.com), Adobe illustrator (Adobe, https://www.adobe.com).

## Discussion

RCC remains a major global health burden, particularly in advanced stages where therapeutic options are limited (Capitanio et al., 2019). Current first-line treatment strategies mainly rely on combinations of targeted therapies and immune checkpoint inhibitors; however, complete remission is achieved in only a small proportion of patients (Braun et al., 2021). These limitations highlight an urgent need to further determine the molecular mechanisms underlying RCC progression and to develop more effective therapeutic agents.

The human body harbors a huge reservoir of bioactive peptides, comprising millions of peptide species, the majority of which are generated through proteolytic processing of precursor proteins. A subset of these peptides exhibits tissue-specific expression patterns and plays a pivotal role in the initiation and progression of various diseases (Muttentha et al., 2021; Lv et al., 2021). Systematic exploration of these endogenous peptides would expand our mechanistic understanding of human pathophysiology and open new paths for therapeutic intervention. Recent studies have identified several endogenous peptides with intrinsic anti-tumor activities. For example, Zhang et al. used peptidomics technology, which led to the discovery of CBDP1,a peptide derived from human Cathepsin B, which suppresses the progression of clear cell renal cell carcinoma by regulating the USP5/YTHDF2/TRPM5 axis (Zhang et al., 2025b). Similarly, Chen et al. identified a novel peptide TCL6148 encoded by TCL6 (T Cell Leukemia/Lymphoma 6), which exerts anti-tumor effects by triggering ferroptosis in RCC cells through the GOT1/GPX4 signaling pathway (Chen Z. et al., 2025). Furthermore, Li et al. unveiled the new peptide PDBAG1 derived from the precursor protein GPD1 by employing a peptidomics-based drug screening strategy. This peptide demonstrated potential efficacy in suppressing triple-negative breast cancer, both *in vitro* and *in vivo* (Li et al., 2025). However, many more bioactive endogenous peptides remain to be discovered.

In our previous work, we adopted a peptidomics-based strategy to identify and synthesize an antitumor peptide, Tat-hspb1. Building upon the same comprehensive peptidomic dataset, the present study identified a 21-amino-acid peptide derived from the N-terminus of the HSPE1 precursor protein. By conjugating this sequence to the cell-penetrating peptide Tat, we generated a novel fusion peptide, Tat-HSPE1. Functional analyses demonstrated that Tat-HSPE1 markedly suppressed ccRCC progression, exhibiting robust antitumor activity both *in vitro* and *in vivo*, while maintaining favorable tumor selectivity and low level toxicity toward normal cells. In terms of the mechanism involved, Tat-HSPE1 acts as a dual inducer of lysosomal membrane permeabilization and autophagy. We further identified CTTNBP2NL, a negative regulator of autophagy and a component of the STRIPAK complex, as a key interacting partner of Tat-HSPE1. The STRIPAK complex has been implicated in tumorigenesis and metastasis, suggesting a functional link between Tat-HSPE1–CTTNBP2NL interaction and tumor suppression. In addition, Tat-HSPE1 was predominantly localized to the nucleolus, indicating that its intracellular distribution may contribute to its biological activity.

Tat, a prototypical cell-penetrating peptide, delivers its cargo into cells mainly through two mechanisms: direct translocation driven by the transmembrane potential and secondly, bu endocytosis, with the dominant pathway depending on the physicochemical properties of the conjugated moiety (Lin and Alexander-Katz, 2013). Guided by the predicted α-helical structure of Tat-HSPE1, it is proposed that Tat-HSPE1 cellular uptake is likely predominantly mediated by endocytosis, followed by endosomal escape into the cytoplasm. After internalization, Tat is recognized by nuclear pore complexes, thereby facilitating the nuclear translocation of Tat-HSPE1 (Jin et al., 2013).

Within the nucleus, Tat-HSPE1 interacts with CTTNBP2NL, leading to activation of autophagy and induction of DNA damage. Notably, DNA damage has been reported to act as an upstream regulator of autophagy (Lascaux et al., 2024), suggesting that Tat-HSPE1–induced genotoxic stress may further potentiate autophagic signaling. In the cytoplasm, Tat-HSPE1 can be delivered to lysosomes either directly or through autophagosome–lysosome fusion during autophagy. This process ultimately results in lysosomal membrane permeabilization, followed by the release of lysosomal hydrolases into the cytosol and irreversible cell death.

Heat shock proteins (HSPs) primarily function as molecular chaperones that regulate protein folding, assembly, translocation, and degradation (Goel et al., 2025). Beyond their canonical roles in proteostasis, HSPs are increasingly recognized as key modulators of cellular metabolism, signal transduction, and programmed cell death (Zuo et al., 2024; Lin et al., 2020). HSPE1 is a highly conserved member of the HSP family across species and serves as a significant component of the cellular protein-folding machinery (Jung et al., 2025; Wardelmann et al., 2021). More importantly, aberrant expression of HSPE1 has been reported in several malignancies, including non-small cell lung cancer and ovarian carcinoma, and has been proposed as a prognostic biomarker (Têtu et al., 2008; Tang et al., 2021). Taken together, despite its relatively small molecular size, HSPE1 exerts multifaceted roles beyond chaperoning, with compelling links to tumorigenesis and tumor immune modulation.

Studies have shown that heat shock proteins purified from specific tumors can trigger specific immunity against that tumor, suggesting that the heat shock protein family is a promising research tool in the field of tumor treatment (Ménoret and Bell, 2000). Together with the findings from our prior investigations on Tat-hspb1 (Zhang et al., 2022), we postulated that the heat shock protein family, along with their derived peptides, may play a critical role in the treatment of RCC. Understanding these mechanisms holds promise for the development of novel, targeted therapeutics for RCC, offering a prospective avenue for significant clinical advancements.

Through integrative analysis of cell death pathway inhibitor assays and proteomic profiling, we demonstrated that Tat-HSPE1 predominantly nduced ccRCC cell death via lysosomal membrane permeabilization. Contrary to our expectations, Tat-HSPE1 exhibited predominant nucleolar localization rather than lysosomal accumulation, where it interacted with CTTNBP2NL to modulate autophagy. While our study identified CTTNBP2NL as a novel autophagy regulator, the precise molecular mechanisms underlying this regulation remain to be determined. Furthermore, the intracellular trafficking, binding dynamics, and degradation of Tat-HSPE1 involve complex regulatory networks, including DNA damage responses, pyroptosis, necroptosis, oxidative phosphorylation, and PPAR signaling pathways. These pathways exhibit extensive crosstalk, with overlapping or hierarchical relationships. Given the multiplicity of Tat-HSPE1 protein interactions, the observed phenotypes cannot be fully explained by any single molecule or linear signaling cascade. Thus, categorizing Tat-HSPE1-induced cell death strictly as lysosome-dependent cell death may represent an oversimplification of its demonstrated pleiotropic mechanisms.

*In vitro* experiments revealed that Tat-HSPE1 exhibited selective toxicity toward renal carcinoma cells, while displaying minimal effects on normal epithelial cell lines and other cancer cell types. To explore the mechanistic basis of this specificity, we focused on the potential interacting proteins. However, CTTNBP2NL did not exhibit unique or differential expression specific to renal cancer cell lines. To provide better clarity, we propose to increase the sample size to include a broader panel of normal epithelial and non-renal cancer cell lines in future studies. Subsequent stratification of cell lines into Tat-HSPE1-sensitive and -insensitive groups, coupled with transcriptome or proteome analysis, may help identify key genetic or protein determinants of this selectivity. Such findings could provide novel targets for precision therapy of RCC.

The cell-penetrating peptide Tat lacks intrinsic targeting specificity, which restricts the clinical applicability of Tat-HSPE1 due to potential off-target effects. Notably, recent studies have demonstrated that amidization of Tat lysine residues to succinyl amides (^a^Tat) effectively abolishes nonspecific interactions in circulation while maintaining stability *in vivo* (Jin et al., 2013). These findings suggest that ^a^Tat-HSPE1 may offer improved therapeutic efficacy and systemic safety. Alternatively, tumor-homing peptides such as RGD or NGR could be incorporated to enhance the tumor-selective delivery of HSPE1 peptide fusion constructs. Such modifications may provide a viable strategy to achieve targeted therapy while minimizing adverse effects.

## Conclusion

**FIGURE 7:**
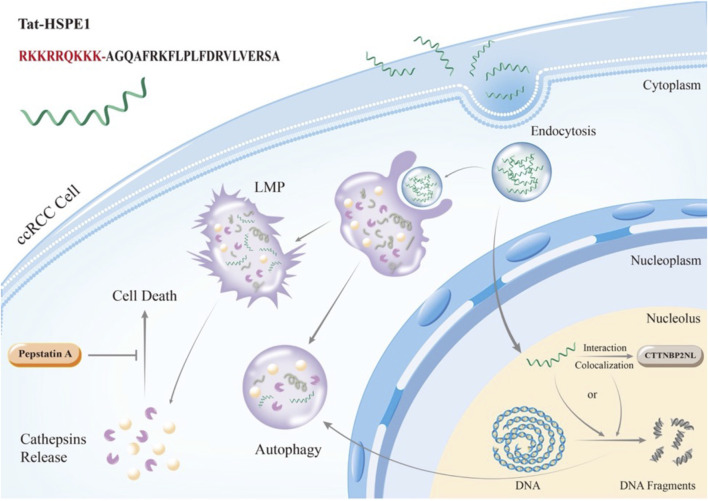
Schematic illustration of the role and molecular mechanisms of Tat-HSPE1 in ccRCC. Tat-HSPE1 inhibits ccRCC progression by interacting with CTTNBP2NL, driving the enhancement of autophagic flux and inducing LMP.

In this study, we identified the fusion peptide Tat-HSPE1 as a potent suppressor of ccRCC. Tat-HSPE1 targets CTTNBP2NL to activate autophagy, leading to lysosomal membrane permeabilization and cathepsin release (Figure 7). This peptide holds promise in informing novel mechanistic understanding and therapeutic approaches for ccRCC.

## Data Availability

The original contributions presented in the study are included in the article/Supplementary Material, further inquiries can be directed to the corresponding authors.

## References

[B1] Ahmad F. Kumar P. Singh P. Joshi T. Singh P. K. (2025). Zika virus impairs autophagic flux in trabecular meshwork, and inhibition of autophagy restricts ocular viral transmission and associated pathology. Microbiol. Spectr. 13, e0103425. 10.1128/spectrum.01034-25 40920489 PMC12502531

[B2] Braun D. A. Bakouny Z. Hirsch L. Flippot R. Van Allen E. M. Wu C. J. (2021). Beyond conventional immune-checkpoint inhibition - novel immunotherapies for renal cell carcinoma. Nat. Rev. Clin. Oncol. 18, 199–214. 10.1038/s41571-020-00455-z 33437048 PMC8317018

[B3] Capitanio U. Bensalah K. Bex A. Boorjian S. A. Bray F. Coleman J. (2019). Epidemiology of renal cell carcinoma. Eur. Urol. 75, 74–84. 10.1016/j.eururo.2018.08.036 30243799 PMC8397918

[B4] Chen O. Vovk O. Polishchuk N. Mayevska O. Shuvayeva G. Demir M. (2025). Adaptation to arginine deprivation leads to a more aggressive, therapy-resistant phenotype in HNSCC cells. Biomolecules 15, 900. 10.3390/biom15060900 40563540 PMC12190576

[B5] Chen Z. Jia X. Meng K. Li W. Wang Y. Cheng S. (2025). A novel peptide TCL6148 induces ferroptosis *via* the GOT1/GPX4 pathway to enhance sunitinib sensitivity in renal cell carcinoma. Int. J. Biol. Macromol. 313, 144242. 10.1016/j.ijbiomac.2025.144242 40379184

[B6] De Mazière A. van der Beek J. van Dijk S. de Heus C. Reggiori F. Koike M. (2022). An optimized protocol for immuno-electron microscopy of endogenous LC3. Autophagy 18, 3004–3022. 10.1080/15548627.2022.2056864 35387562 PMC9673964

[B7] Dell'Atti L. Bianchi N. Aguiari G. (2022). New therapeutic interventions for kidney carcinoma: looking to the future. Cancers 14 (15), 3616. 10.3390/cancers14153616 35892875 PMC9332391

[B8] Galluzzi L. Vitale I. Aaronson S. A. Abrams J. M. Adam D. Agostinis P. (2018). Molecular mechanisms of cell death: recommendations of the nomenclature committee on cell death 2018. Cell. Death Differ. 25, 486–541. 10.1038/s41418-017-0012-4 29362479 PMC5864239

[B9] Gao X. Yuan C. Tan E. Li Z. Cheng Y. Xiao J. (2023). Dual-responsive bioconjugates bearing a bifunctional adaptor for robust cytosolic peptide delivery. J. Control. Release Official J. Control. Release Soc. 355, 675–684. 10.1016/j.jconrel.2023.02.014 36791993

[B10] Goel B. Jaiswal S. Tripathi N. (2025). Recent advances in HSP90 inhibitors as targeted cancer therapy: chemical scaffolds, isoform selectivity, and clinical progress. Bioorg. Chem. 163, 108782. 10.1016/j.bioorg.2025.108782 40706543

[B11] Goudreault M. D'Ambrosio L. M. Kean M. J. Mullin M. J. Larsen B. G. Sanchez A. (2008). A PP2A phosphatase high density interaction network identifies a novel striatin-interacting phosphatase and kinase complex linked to the cerebral cavernous malformation 3 (CCM3) protein. Mol. and Cell. Proteomics MCP 8, 157–171. 10.1074/mcp.M800266-MCP200 18782753 PMC2621004

[B12] Hamley I. W. (2017). Small bioactive peptides for biomaterials design and therapeutics. Chem. Rev. 117, 14015–14041. 10.1021/acs.chemrev.7b00522 29227635

[B13] Iosub-Amir A. Bai F. Sohn Y. S. Song L. Tamir S. Marjault H. B. (2019). The anti-apoptotic proteins NAF-1 and iASPP interact to drive apoptosis in cancer cells. Chem. Sci. 10, 665–673. 10.1039/c8sc03390k 30774867 PMC6349067

[B14] Jantas D. Chwastek J. Grygier B. Lasoń W. (2020). Neuroprotective effects of Necrostatin-1 against oxidative stress-induced cell damage: an involvement of cathepsin D inhibition. Neurotox. Res. 37, 525–542. 10.1007/s12640-020-00164-6 31960265 PMC7062871

[B15] Jin E. Zhang B. Sun X. Zhou Z. Ma X. Sun Q. (2013). Acid-active cell-penetrating peptides for *in vivo* tumor-targeted drug delivery. J. Am. Chem. Soc. 135, 933–940. 10.1021/ja311180x 23253016

[B16] Jin S. Yan M. Liu Y. Zhang S. Song H. Cao C. (2025). RETREG1-mediated reticulophagy is activated by an ATF4-CEBPG/C/EBPγ heterodimer and confers protection against lipotoxicity. Autophagy 21 (12), 2614–2632. 10.1080/15548627.2025.2512884 40437698 PMC12758220

[B17] Jung M. Kim M. Ham S. J. Chung J. Roh S.-H. (2025). *In situ* characterization of mitochondrial Hsp60-Hsp10 chaperone complex under folding stress. Sci. Adv. 11, eadw6064. 10.1126/sciadv.adw6064 41124257 PMC12542933

[B18] Kaczynski T. J. Husami N. J. Au E. D. Farkas M. H. (2023). Dysregulation of a lncRNA within the TNFRSF10A locus activates cell death pathways. Cell. Death Discov. 9, 242. 10.1038/s41420-023-01544-5 37443108 PMC10344863

[B19] Khalkhali-Ellis Z. Hendrix M. J. C. (2014). Two faces of cathepsin D: physiological guardian angel and pathological demon. Biol. Med. (Aligarh) 6 (2), 1000206. 10.4172/0974-8369.1000206 25663755 PMC4318633

[B20] Larcher A. Campi R. Bex A. Bray F. Bukavina L. Jonasch E. (2025). Epidemiology of renal cancer: incidence, mortality, survival, genetic predisposition, and risk factors. Eur. Urol. 88, 341–358. 10.1016/j.eururo.2025.06.005 40750496

[B21] Lascaux P. Hoslett G. Tribble S. Trugenberger C. Antičević I. Otten C. (2024). TEX264 drives selective autophagy of DNA lesions to promote DNA repair and cell survival. Cell. 187, 5698–5718.e26. 10.1016/j.cell.2024.08.020 39265577

[B22] Li X. Wu Y. Zhang M. Wang F. Yin H. Zhang Y. (2025). A new peptide inhibitor of C1QBP exhibits potent anti-tumour activity against triple negative breast cancer by impairing mitochondrial function and suppressing homologous recombination repair. Clin. Transl. Med. 15, e70162. 10.1002/ctm2.70162 39748215 PMC11695203

[B23] Lin J. Alexander-Katz A. (2013). Cell membranes open “doors” for cationic nanoparticles/biomolecules: insights into uptake kinetics. ACS Nano 7, 10799–10808. 10.1021/nn4040553 24251827

[B24] Lin T.-Y. Hua W.-J. Yeh H. Tseng A.-J. (2020). Functional proteomic analysis reveals that fungal immunomodulatory protein reduced expressions of heat shock proteins correlates to apoptosis in lung cancer cells. Phytomedicine Int. J. Phytotherapy Phytopharm. 80, 153384. 10.1016/j.phymed.2020.153384 33113507

[B25] Lv S. Sylvestre M. Prossnitz A. N. Yang L. F. Pun S. H. (2021). Design of polymeric carriers for intracellular peptide delivery in oncology applications. Chem. Rev. 121, 11653–11698. 10.1021/acs.chemrev.0c00963 33566580

[B26] Maani Z. Rahbarnia L. Bahadori A. Chollou K. M. Farajnia S. (2024). Spotlight on HIV-Derived TAT peptide as a molecular shuttle in drug delivery. Drug Discov. Today 29, 104191. 10.1016/j.drudis.2024.104191 39322176

[B27] Menjivar N. G. Gad A. Thompson R. E. Meyers M. A. Ghosh S. Hollinshead F. K. (2025). Organoids simulating the bovine oviduct mediate the embryo-maternal interface *via* extracellular vesicle-transmitted signaling. Hum. Reprod. Open 2026, hoaf076. 10.1093/hropen/hoaf076 41503164 PMC12774516

[B28] Ménoret A. Bell G. (2000). Purification of multiple heat shock proteins from a single tumor sample. J. Immunol. Methods 237, 119–130. 10.1016/s0022-1759(00)00137-x 10725457

[B29] Min K. J. Kwon T. K. (2020). Induction of lysosomal membrane permeabilization is a major event of FTY720-Mediated non-apoptotic cell death in human glioma cells. Cancers 12 (11), 3388. 10.3390/cancers12113388 33207629 PMC7696845

[B30] Motzer R. J. Jonasch E. Agarwal N. Alva A. Bagshaw H. Baine M. (2024). NCCN guidelines® insights: Kidney cancer, version 2.2024. J. Natl. Compr. Cancer Netw. JNCCN 22, 4–16. 10.6004/jnccn.2024.0008 38394781

[B31] Mukherjee A. G. Wanjari U. R. Gopalakrishnan A. V. Bradu P. Biswas A. Ganesan R. (2023). Evolving strategies and application of proteins and peptide therapeutics in cancer treatment. Biomed. and Pharmacother. = Biomedecine and Pharmacother. 163, 114832. 10.1016/j.biopha.2023.114832 37150032

[B32] Muttenthaler M. King G. F. Adams D. J. Alewood P. F. (2021). Trends in peptide drug discovery. Nat. Rev. Drug Discov. 20, 309–325. 10.1038/s41573-020-00135-8 33536635

[B33] Netzer A. Katzir I. Baruch Leshem A. Weitman M. Lampel A. (2023). Emergent properties of melanin-inspired peptide/RNA condensates. Proc. Natl. Acad. Sci. U. S. A. 120, e2310569120. 10.1073/pnas.2310569120 37871222 PMC10622964

[B34] Norell P. N. Campisi D. Mohan J. Wollert T. (2024). Biogenesis of omegasomes and autophagosomes in mammalian autophagy. Biochem. Soc. Trans. 52, 2145–2155. 10.1042/BST20240015 39392358 PMC11555699

[B35] Roberts R. Ktistakis N. T. (2013). Omegasomes: PI3P platforms that manufacture autophagosomes. Essays Biochem. 55, 17–27. 10.1042/bse0550017 24070468

[B36] Seo G. McKinley J. Wang W. (2024). MAP4K2 connects the hippo pathway to autophagy in response to energy stress. Autophagy 20, 704–706. 10.1080/15548627.2023.2280876 37937799 PMC10936684

[B37] Shao R. Liu W. Feng Y. Guo X. Ren Z. Hou X. (2025). LAMP2-FLOT2 interaction enhances autophagosome-lysosome fusion to protect the septic heart in response to ILC2. Autophagy 21 (9), 1888–1910. 10.1080/15548627.2025.2469207 40066518 PMC12366814

[B38] Shi J. Zhao Y. Wang K. Shi X. Wang Y. Huang H. (2015). Cleavage of GSDMD by inflammatory caspases determines pyroptotic cell death. Nature 526, 660–665. 10.1038/nature15514 26375003

[B39] Shimizu N. Kanemitsu S. Umemura R. Yashiro T. Kawabata R. Nishimura K. (2025). Mechanistic insights into the apoptosis of cancer cells induced by a kinase-responsive peptide amphiphile. Chem. Weinheim Der Bergstrasse, Ger. 31, e202403658. 10.1002/chem.202403658 39876747

[B40] Shoji-Kawata S. Sumpter R. Leveno M. Campbell G. R. Zou Z. Kinch L. (2013). Identification of a candidate therapeutic autophagy-inducing peptide. Nature 494, 201–206. 10.1038/nature11866 23364696 PMC3788641

[B41] Tang Y. Yang Y. Luo J. Liu S. Zhan Y. Zang H. (2021). Overexpression of HSP10 correlates with HSP60 and Mcl-1 levels and predicts poor prognosis in non-small cell lung cancer patients. Cancer Biomarkers Sect. A Dis. Markers 30, 85–94. 10.3233/CBM-200410 32986659 PMC7990427

[B42] Tasdemir E. Chiara Maiuri M. Morselli E. Criollo A. D'Amelio M. Djavaheri-Mergny M. (2008). A dual role of p53 in the control of autophagy. Autophagy 4, 810–814. 10.4161/auto.6486 18604159

[B43] Têtu B. Popa I. Bairati I. L'Esperance S. Bachvarova M. Plante M. (2008). Immunohistochemical analysis of possible chemoresistance markers identified by micro-arrays on serous ovarian carcinomas. Mod. Pathology An Official J. U. S. Can. Acad. Pathology, Inc 21, 1002–1010. 10.1038/modpathol.2008.80 18500265

[B44] Valentini S. Mele G. Attili M. Assenza M. R. Saccoccia F. Sardina F. (2025). Targeting the MDM2-MDM4 interaction interface reveals an otherwise therapeutically active wild-type p53 in colorectal cancer. Mol. Oncol. 19, 2412–2430. 10.1002/1878-0261.70006 40022459 PMC12330940

[B45] Waisner H. Lasnier S. Suma S. M. Kalamvoki M. (2023). Effects on exocytosis by two HSV-1 mutants unable to block autophagy. J. Virology 97, e0075723. 10.1128/jvi.00757-23 37712703 PMC10617559

[B46] Wang L.-H. Wei S. Yuan Y. Zhong M.-J. Wang J. Yan Z.-X. (2024). KPT330 promotes the sensitivity of glioblastoma to olaparib by retaining SQSTM1 in the nucleus and disrupting lysosomal function. Autophagy 20, 295–310. 10.1080/15548627.2023.2252301 37712615 PMC10813631

[B47] Wardelmann K. Rath M. Castro J. P. Blümel S. Schell M. Hauffe R. (2021). Central acting Hsp10 regulates mitochondrial function, fatty acid metabolism, and insulin sensitivity in the hypothalamus. Antioxidants Basel, Switz. 10, 711. 10.3390/antiox10050711 33946318 PMC8145035

[B48] Xu J. Yang K. C. Go N. E. Colborne S. Ho C. J. Hosseini-Beheshti E. (2022). Chloroquine treatment induces secretion of autophagy-related proteins and inclusion of Atg8-family proteins in distinct extracellular vesicle populations. Autophagy 18, 2547–2560. 10.1080/15548627.2022.2039535 35220892 PMC9629075

[B49] Yang J. Liu Z. Wang C. Yang R. Rathkey J. K. Pinkard O. W. (2018). Mechanism of gasdermin D recognition by inflammatory caspases and their inhibition by a gasdermin D-derived peptide inhibitor. Proc. Natl. Acad. Sci. U. S. A. 115, 6792–6797. 10.1073/pnas.1800562115 29891674 PMC6042100

[B50] Zein L. Dietrich M. Balta D. Bader V. Scheuer C. Zellner S. (2025). Linear ubiquitination at damaged lysosomes induces local NFKB activation and controls cell survival. Autophagy 21, 1075–1095. 10.1080/15548627.2024.2443945 39744815 PMC12013452

[B51] Zhang N. Yang Y. Wang Z. Yang J. Chu X. Liu J. (2019). Polypeptide-engineered DNA tetrahedrons for targeting treatment of colorectal cancer *via* apoptosis and autophagy. J. Control. Release Official J. Control. Release Soc. 309, 48–58. 10.1016/j.jconrel.2019.07.012 31301339

[B52] Zhang L. Jin G.-Z. Li D. (2022). Tat-hspb1 suppresses clear cell renal cell carcinoma (ccRCC) growth *via* lysosomal membrane permeabilization. Cancers 14, 5710. 10.3390/cancers14225710 36428802 PMC9688814

[B53] Zhang Y. Cai W. Wu A. Li R. Li X. Zheng K. (2025a). Necrostatin-1 alleviates temporomandibular joint osteoarthritis and inhibits chondrocyte senescence and necroptosis possibly *via* p53/MAPK downregulation. Front. Bioeng. Biotechnol. 13, 1735484. 10.3389/fbioe.2025.1735484 41487948 PMC12756420

[B54] Zhang Y. Zhu W. Tao R. Li W. Jiang C. Yan X. (2025b). Endogenous peptide CBDP1 inhibits clear cell renal cell carcinoma progression by targeting USP5/YTHDF2/TRPM5 axis. J. Transl. Med. 23, 116. 10.1186/s12967-025-06091-4 39863860 PMC11763126

[B55] Zhang Z. Zhang M. Cao Z. Zhao H. Li X. Luo P. (2026). Fibrillarin: bridging ribosome biogenesis and apoptosis in cellular stress and disease. Apoptosis An Int. J. Program. Cell. Death 31, 11. 10.1007/s10495-025-02220-y 41518572

[B56] Zhao C. Miao D. Tan D. Shi J. Lv Q. Xiong Z. (2024). The PLCG2 inhibits tumor progression and mediates angiogenesis by VEGF signaling pathway in clear cell renal cell carcinoma. Front. Biosci. Landmark Ed. 29, 390. 10.31083/j.fbl2911390 39614428

[B57] Zhou W. Lim A. Edderkaoui M. Osipov A. Wu H. Wang Q. (2024). Role of YAP signaling in regulation of programmed cell death and drug resistance in cancer. Int. J. Biol. Sci. 20, 15–28. 10.7150/ijbs.83586 38164167 PMC10750275

[B58] Zhu J. Yang Y. Wu J. (2007). Bcl-2 cleavages at two adjacent sites by different caspases promote cisplatin-induced apoptosis. Cell. Res. 17, 441–448. 10.1038/cr.2007.36 17452997

[B59] Zuo W.-F. Pang Q. Zhu X. Yang Q.-Q. Zhao Q. He G. (2024). Heat shock proteins as hallmarks of cancer: insights from molecular mechanisms to therapeutic strategies. J. Hematol. and Oncol. 17, 81. 10.1186/s13045-024-01601-1 39232809 PMC11375894

