# Supplementary material for: Tat-HSPE1 suppresses clear cell renal cell carcinoma growth through lysosome-dependent cell death
Source: Front Pharmacol. 2026 May 1;17:1822208. doi: 10.3389/fphar.2026.1822208 (PMC13176256; doi:10.3389/fphar.2026.1822208)
Supplement: Supplementary file 1 [file Supplementaryfile1.docx]

Tat-HSPE1 suppresses clear cell renal cell carcinoma growth through lysosome-dependent cell death

Lin Zhang, Weiyuan Li, Min Liu, Dong Li, and Guang-Zhi Jin

Supplemental information


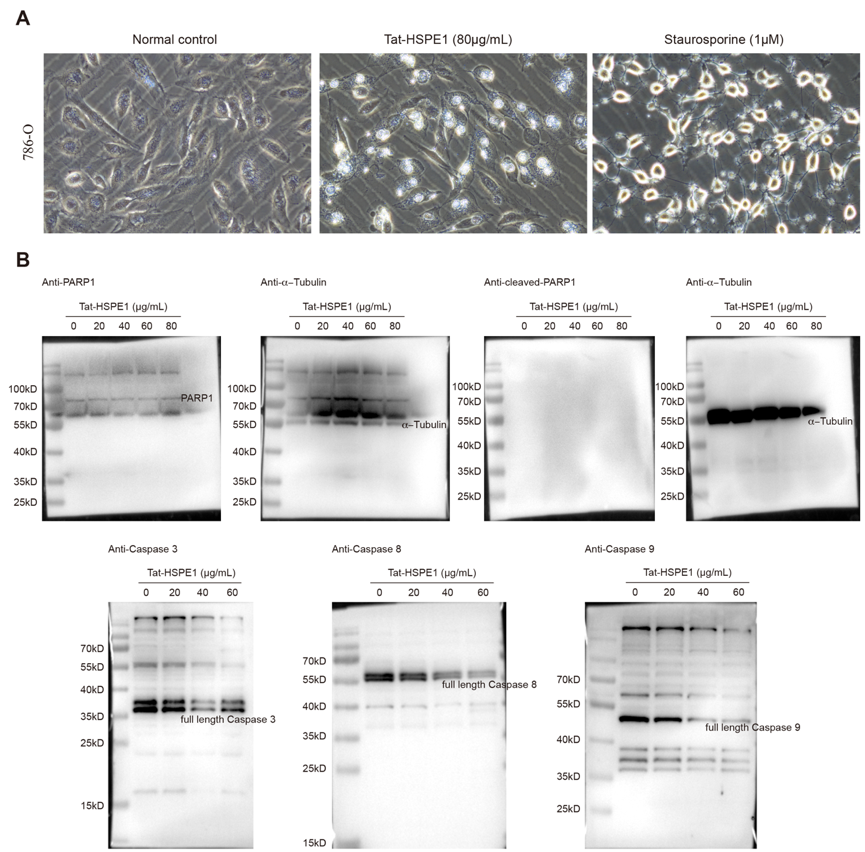


Figure S1. Tat-HSPE1 induces cell death of ccRCC, related to figure 2

(A) 786-O cells were treated with Tat-HSPE1 and Staurosporine, bright-field microscopy shows obviously different morphological changes.

(B) 786-O cells were treated with indicated concentrations of Tat-HSPE1, the expression of PARP1, cleaved-PARP1, caspase-3, caspase-8 and caspase-9 were measured with western blot.


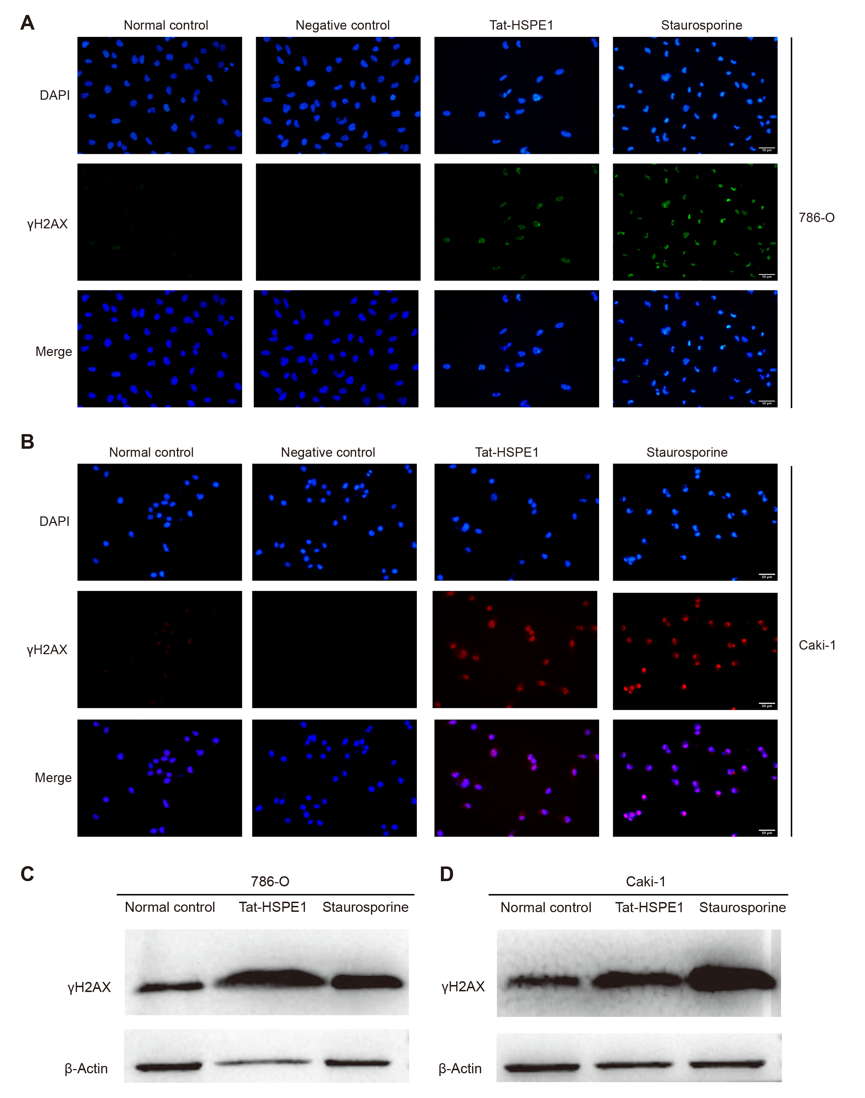


Figure S2. Tat-HSPE1 induces DNA damage of ccRCC, related to figure 2

(A and B) DNA damage in ccRCC cells. Immunofluorescence analysis of γH2AX in 786-O and Caki-1 cells treated with saline, Tat-HSPE1 and staurosporine. Staurosporine was used as a positive control.

(C and D) Western blot analysis of protein levels of γH2AX in the control, Tat-HSEP1 and staurosporine treated 786-O (C) and Caki-1 (D) cells. β-Actin was shown as a loading control.


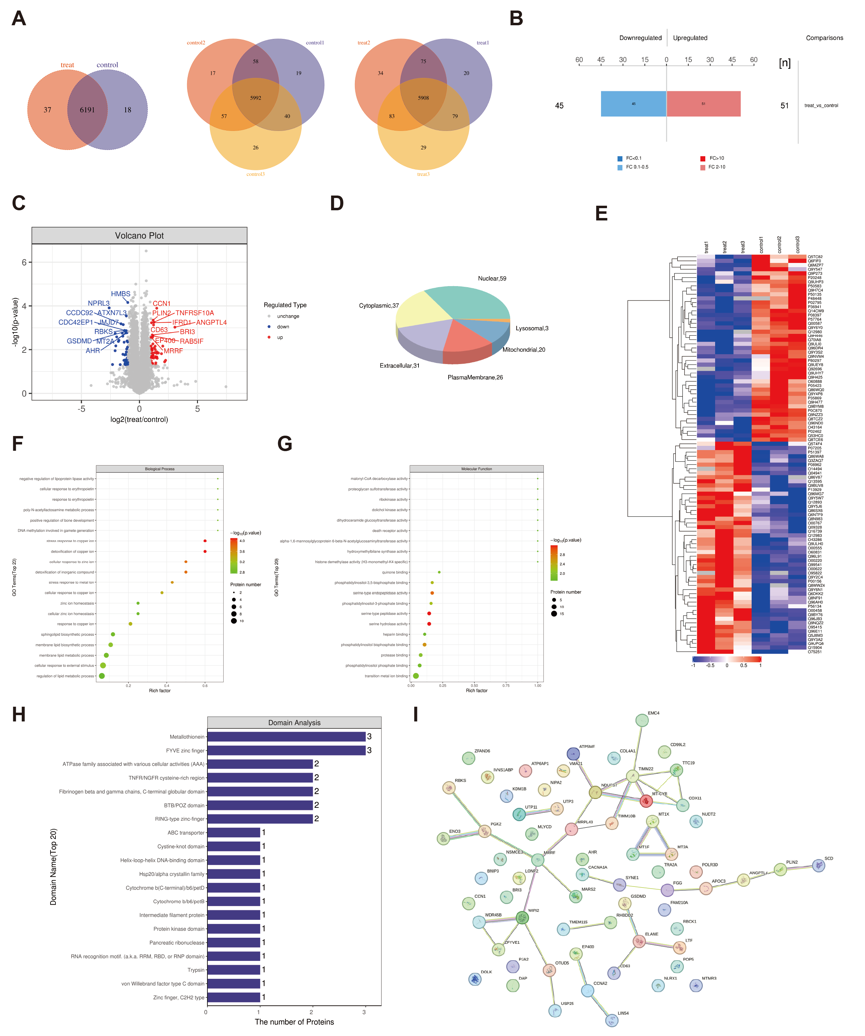


Figure S3. The Proteomics reveals changes in the protein profile after Tat-HSPE1 treatment, related to figure 4

(A) The Venn diagram of the total protein identified through proteomics as well as the overlap of proteins between and within Tat-HSPE1–treated group and control group.

(B, C and E) Histogram, volcano plot and cluster heatmap of differentially expressed proteins after Tat-HSPE1 treatment.

(D) Subcellular localization of differentially expressed proteins in 786-O cells after Tat-HSPE1 treatment.

(F, G and H) Top 20 enrichment terms of biological process, molecular function and protein domain of differentially expressed proteins in 786-O cells after Tat-HSPE1 treatment.

(I) Protein–protein interaction network analysis of differentially expressed proteins in 786-O cells after Tat-HSPE1 treatment based on STRING (https://cn.string-db.org/).


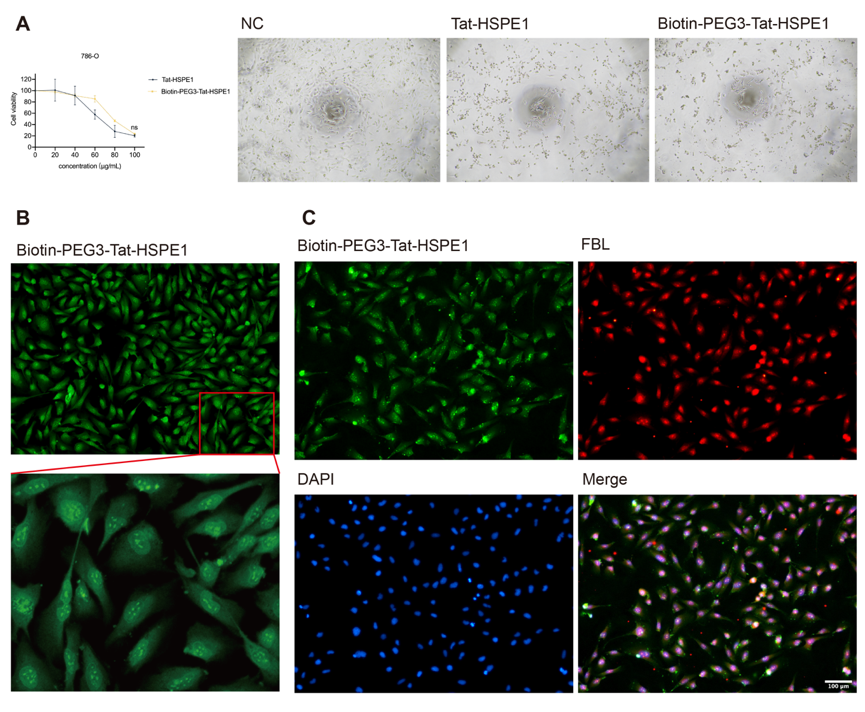


Figure S4. The activity and subcellular localization of Biotin-PEG3-Tat-HSPE1, related to figure 5

(A) Cell viability of 786-O cells treated with saline, Tat-HSPE1 and Biotin-PEG3-Tat-HSPE1.

(B) Subcellular localization of Biotin-PEG3-Tat-HSPE1 in 786-O cells detected by immunofluorescence staining.

(C) IF assay showing localization of Biotin-PEG3-Tat-HSPE1 and FBL in 786-O cells.


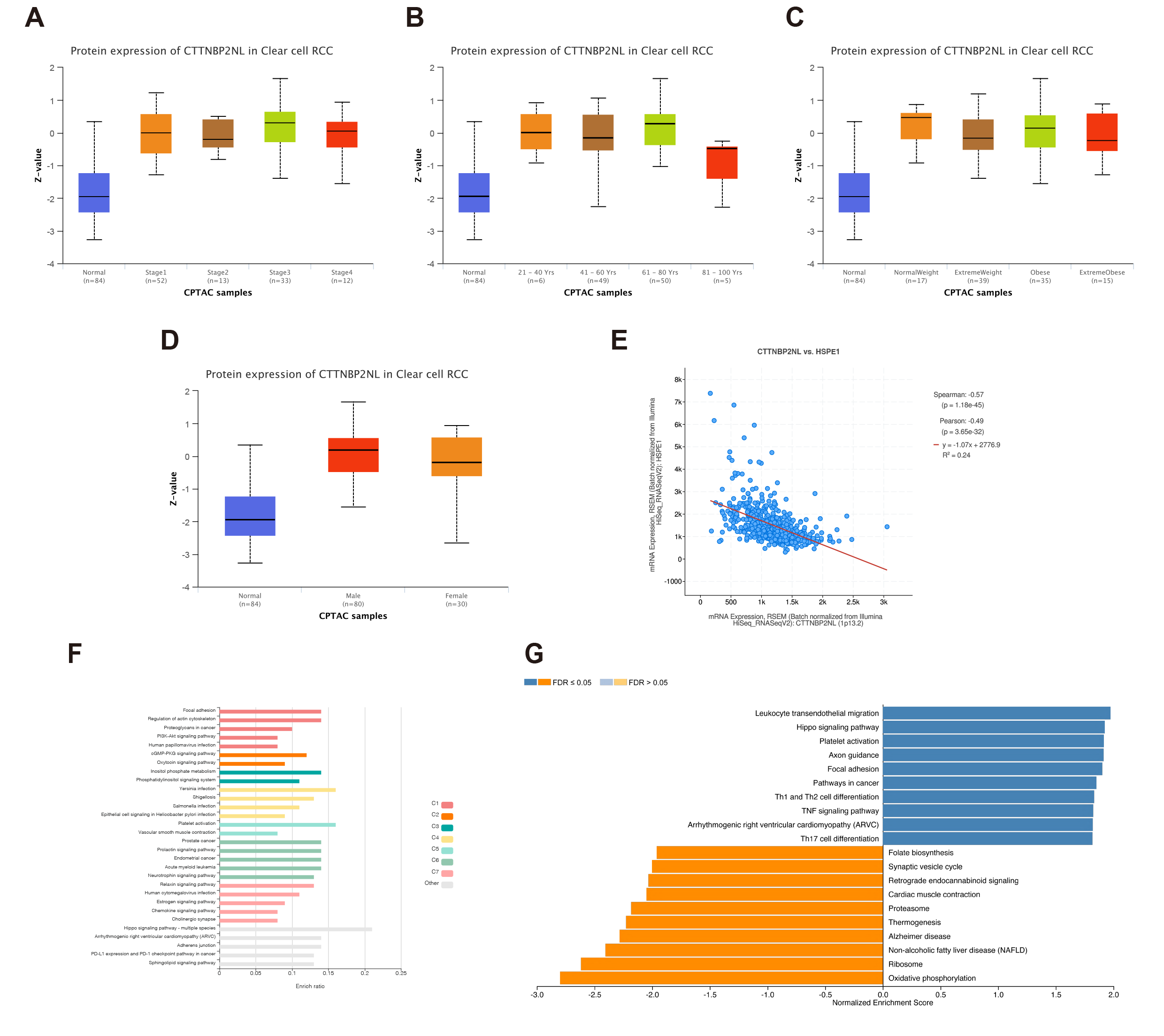


Figure S5. Bioinformatic analysis of CTTNBP2NL in ccRCC, related to figure 5

(A–D) Differential expression of CTTNBP2NL protein among ccRCC in different stages, weights, ages and sexes accessed by the UALCAN database (https://ualcan.path.uab.edu/analysis-prot.html).

(E) Pearson and Spearman correlation analyses were employed to evaluate the co-expression of CTTNBP2NL and HSPE1 in ccRCC using the cBioPortal (https://www.cbioportal.org/).

(F) CTTNBP2NL-related pathways enrichment analysis accessed by the KOBAS database (<http://bioinfo.org/kobas>).

(G) CTTNBP2NL-related pathways enrichment analysis accessed by the Linkedomics database (https://www.linkedomics.org/).

Table S1. Differentially expressed peptides in paired ccRCC and adjacent normal tissues identified by peptidomics analysis, related to Figure 4.


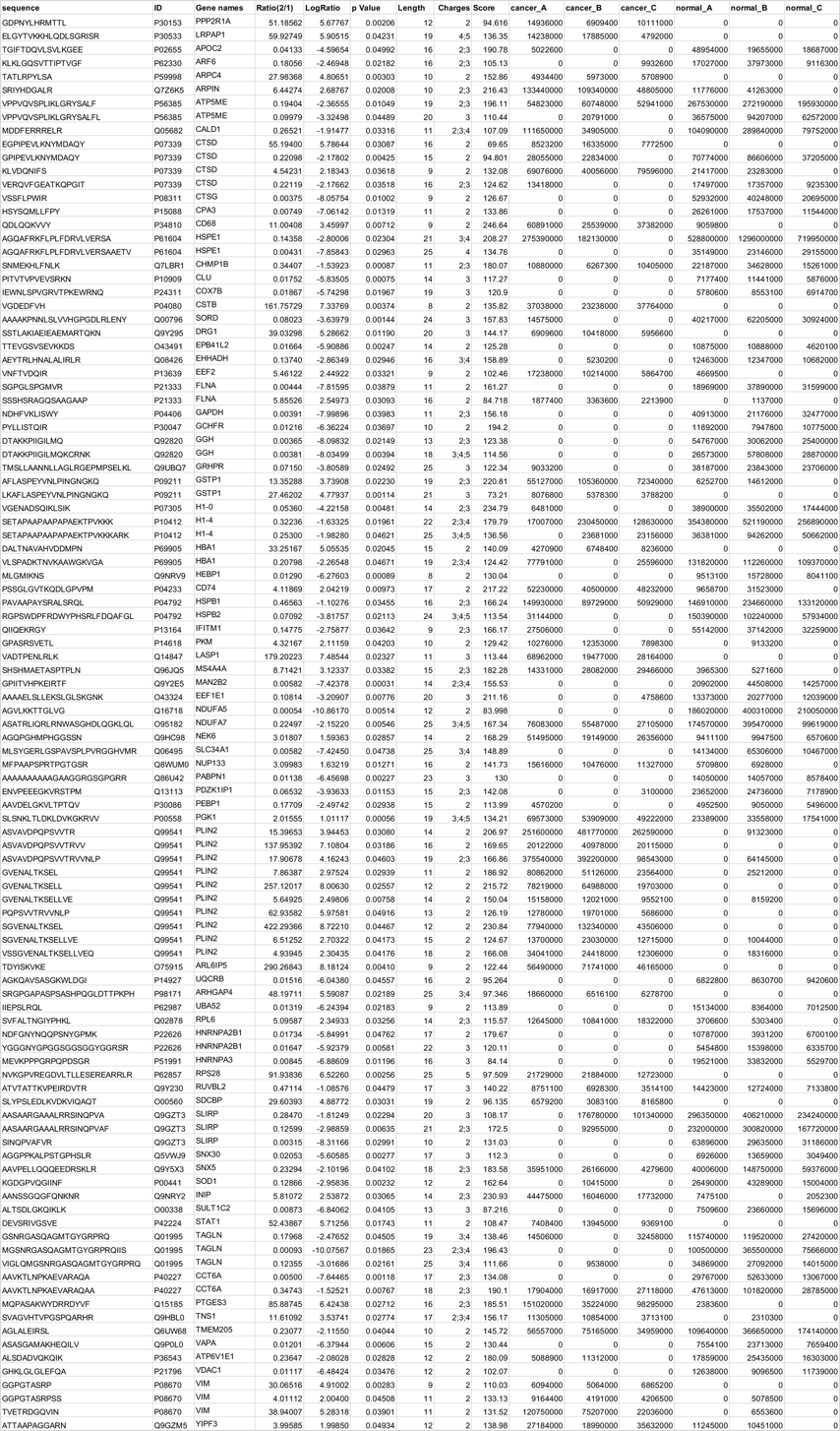


Table S2. Differentially expressed proteins in 786-O cells after Tat-HSPE1 treatment, related to Figure 4.


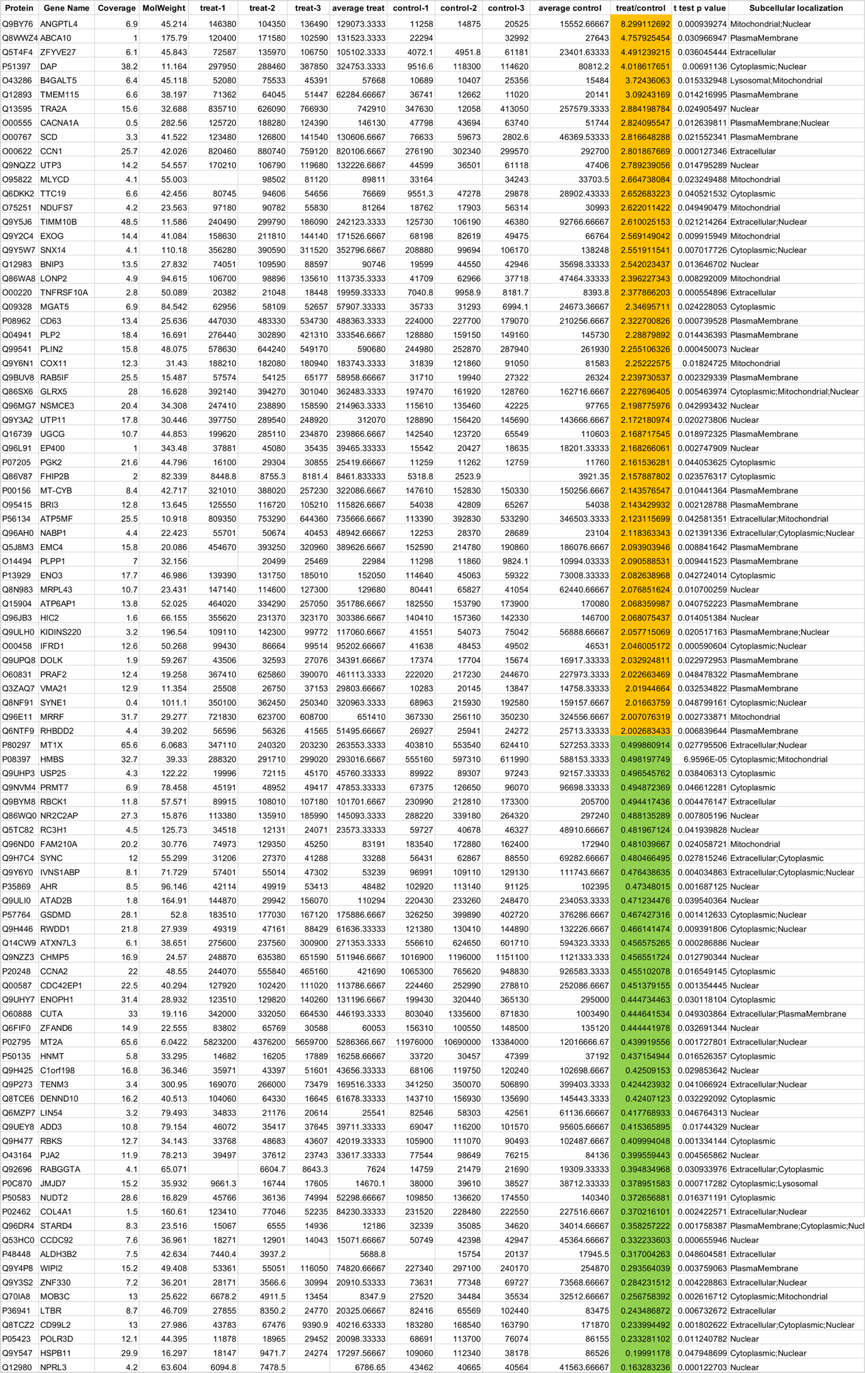


Table S3. Proteins only identified in the Tat-HSPE1–treated group or the control group, related to Figure 4.


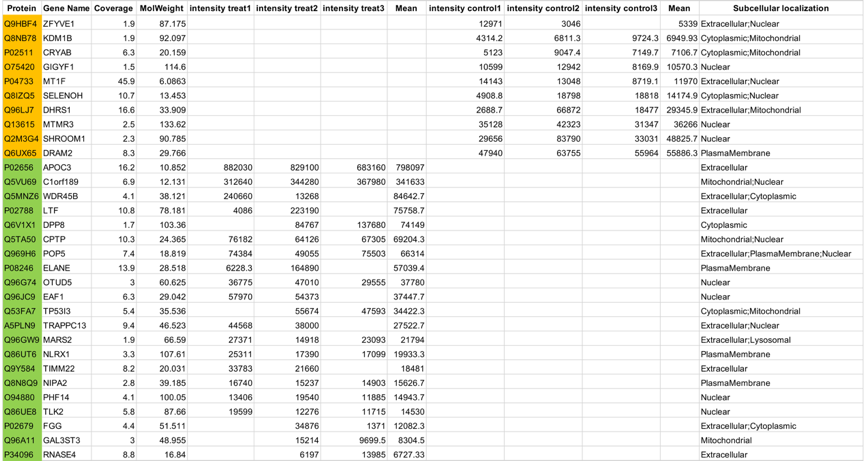


# Table S4. After affinity column chromatography, the top 20 most abundant proteins identified by LC-MS/MS, related to Figure 5.


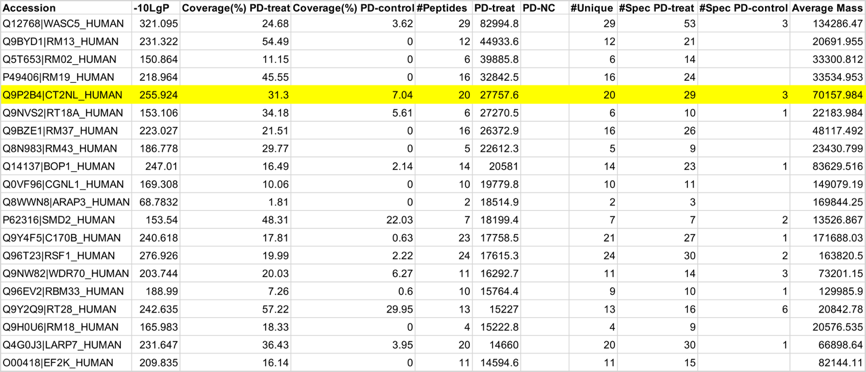


Table S5. siRNA for CTTNBP2NL, related to Figure 5.

| Target | Sequence (Sense strand) | Sequence (Antisense strand) |
| --- | --- | --- |
| CTTNBP2NL (si-NC)  CTTNBP2NL (si-1)  CTTNBP2NL (si-2) | UUCUCCGAACGUGUCACGUTT  CUUUCAUUGAAGAACGCUATT  GAAGAAAUGGAAAGUUUAATT | ACGUGACACGUUCGGAGAATT  UAGCGUUCUUCAAUGAAAGTT  UUAAACUUUCCAUUUCUUCTT |
